# Uncovering a new family of conserved virulence factors that promote the production of host‐damaging outer membrane vesicles in gram‐negative bacteria

**DOI:** 10.1002/jev2.70032

**Published:** 2025-01-22

**Authors:** Audrey Goman, Bérengère Ize, Katy Jeannot, Camille Pin, Delphine Payros, Cécile Goursat, Léa Ravon‐Katossky, Kazunori Murase, Camille V. Chagneau, Hélène Revillet, Frédéric Taieb, Sophie Bleves, Laure David, Etienne Meunier, Priscilla Branchu, Eric Oswald

**Affiliations:** ^1^ Institut de Recherche en Santé Digestive (IRSD) Université de Toulouse, INSERM, INRAE, ENVT, UPS Toulouse France; ^2^ Laboratoire d'Ingénierie des Systèmes Macromoléculaires (LISM‐UMR7255), Institut de Microbiologie de la Méditerannée (IMM) Aix‐Marseille Université, Centre National de la Recherche Scientifique Marseille France; ^3^ Centre National de Référence de la Résistance aux Antibiotiques Centre Hospitalier Universitaire de Besançon Besançon France; ^4^ Institut de Pharmacologie et de Biologie Structurale (IPBS) Université de Toulouse, CNRS, UPS Toulouse France; ^5^ Department of Microbiology, Graduate School of Medicine Kyoto University Kyoto Japan; ^6^ Service de Bactériologie‐Hygiène Centre Hospitalier Universitaire de Toulouse, Hôpital Purpan Toulouse France

**Keywords:** autophagy, inflammasome, OMVs, pathogenicity, PmrAB, polymyxins, Pseudomonas aeruginosa

## Abstract

CprA is a short‐chain dehydrogenase/reductase (SDR) that contributes to resistance against colistin and antimicrobial peptides. The *cprA* gene is conserved across *Pseudomonas aeruginosa* clades and its expression is directly regulated by the two‐component system PmrAB. We have shown that *cprA* expression leads to the production of outer membrane vesicles (OMVs) that block autophagic flux and have a greater capacity to activate the non‐canonical inflammasome pathway. In a murine model of sepsis, a *P. aeruginosa* strain deleted for *cprA* was less virulent than the wild‐type (WT) strain. These results demonstrate the important role of CprA in the pathogenicity of *P. aeruginosa*. It is worth noting that CprA is also a functional ortholog of hemolysin F (HlyF), which is encoded by virulence plasmids of *Escherichia coli*. We have shown that other cryptic SDRs encoded by mammalian and plant pathogens, such as *Yersinia pestis* and *Ralstonia solanacearum* are functional orthologs of CprA and HlyF. These SDRs also induce the production of OMVs which block autophagic flux. This study uncovers a new family of virulence determinants in Gram‐negative bacteria, offering potential for innovative therapeutic interventions and deeper insights into bacterial pathogenesis.

## 1 INTRODUCTION

*Pseudomonas aeruginosa* is a leading cause of chronic and healthcare‐associated infections, exhibiting alarming levels of antimicrobial resistance, morbidity and mortality (Qin et al., 2022). Contributing significantly to global bacterial fatalities, *P. aeruginosa* ranks amongst the top five pathogens, accounting for half of these deaths (Ikuta, 2022). The escalation of this threat is propelled by the pathogen's remarkable ability to develop resistance through chromosomal mutations and the rising prevalence of transferable resistance determinants (López‐Causapé et al., 2018). Annually, over 300,000 deaths are attributed to antibiotic‐resistant *P. aeruginosa* infections, warranting urgent attention from the WHO for novel research and development (Murray et al., 2022).

Polymyxins (i.e., polymyxin B and colistin), represent a crucial class of cyclic lipopeptides antibiotics, that maintain efficacy against extensively‐drug resistant *P. aeruginosa* (Nang et al., 2021). Amongst the proteins involved in polymyxin resistance, PA1559, also known as CprA (for cationic peptide resistance), emerges as a hypothetical protein playing a role in *P. aeruginosa* resistance to polymyxins and antimicrobial peptides (Gutu et al., 2015). The expression of *cprA* is positively regulated by the two‐component system PmrAB, a key regulator of LPS modifications involved in polymyxin resistance in *P. aeruginosa* (Mcphee et al., 2006), but also indirectly by other two‐component systems such as ParRS, CprRS and PhoPQ (Fernández et al., 2012; Gooderham et al., 2009; Muller et al., 2011). Activation of PmrB is triggered by various signals including polymyxins, antimicrobial peptides and low Mg^2+^ (Mcphee et al., 2006; Moskowitz et al., 2004).

The predicted CprA structure is typical of an extended short‐chain dehydrogenase/reductase (SDR) family member (Kavanagh et al., 2008). Despite this insight, the function and substrate of CprA remain unknown. However, CprA exhibits homology with *E. coli* hemolysin F (HlyF), a protein which is encoded by a virulence plasmid found in various *E. coli* pathotypes and in *Salmonella enterica* serovar Kentucky (Chagneau et al., 2023; Murase et al., 2016). It is important to note that HlyF itself lacks hemolytic activity but instead acts as a cytoplasmic enzyme triggering the formation of outer membrane vesicles (OMVs), capable of delivering ClyA, a *bona fide* hemolysin (Murase et al., 2016). The initial assumption that HlyF was a hemolysin was based on this phenotype (Morales et al., 2004). Recently, we demonstrated that HlyF induces the formation of OMVs that not only transport various toxins but also have the intrinsic ability to block autophagic flux and exacerbate inflammasome activation in host cells (David et al., 2022; Finethy et al., 2017; Santos et al., 2018; Vanaja et al., 2016). Here, our findings demonstrate that CprA from *P. aeruginosa* exhibits similar properties to HlyF. Furthermore, our study reveals that functional HlyF/CprA orthologs are also encoded by various Gram‐negative bacteria. This constitutes a new family of virulence factors.

## 2 MATERIALS AND METHODS

### 2.1 Bacterial strains and growth conditions

The strains used in the study were listed in Table S1. Bacteria were routinely grown in lysogeny broth (LB, Lennox). For OMVs production, bacteria were grown in Terrific broth (TB) (Gibco) or in M63 minimal medium (Bio Basic) supplemented with 0.5% Bacto casamino acids (ThermoFisher), 0.2% D+glucose solution and MgCl_2_ (0.1 or 2 mM) (Sigma Aldrich). For plasmid maintenance in *E. coli*, antibiotics were used at the following concentrations: 50 mg/L kanamycin sulfate, 20 mg/L gentamicin, 10 mg/L tetracycline and 30 mg/L streptomycin. For plasmid maintenance in *P. aeruginosa*, antibiotics were used at 500 mg/L streptomycin, 50 mg/L tetracycline, 20 mg/L gentamicin for plasmid. 10 mM L‐arabinose (L‐Ara) (Sigma Aldrich) was added to the bacterial culture for induction of *cprA* under the arabinose inducible promoter and 6 mM L‐rhamnose (Sigma Aldrich) was added to the bacterial culture for the induction of *cprA* under the rhamnose inducible promoter.

### 2.2 Plasmid construction

All the plasmids used in this study are listed in Table S1. Genomic DNA was extracted using the Wizard genomic DNA purification kit (Promega Corporation, Charbonnières‐les‐Bains, France) before amplification by PCR. Expression plasmids used in *E. coli* were built from plasmid pK184 using the NEB HiFi cloning Kit (NEB), with the oligonucleotides listed in Table S2. The plasmid pAGO‐15 was first constructed by the insertion of a PCR product within the *Eco*RI and *Sal*I restriction sites of pK184. The insert of pAGO‐15 contains the intergenic region (432 pb) upstream of the start codon of the *hlyF* ORF and a translational fusion of a 6‐His and S‐tag at the N‐terminus of *hlyF*. The upstream intergenic region and the *hlyF* ORF were PCR‐amplified with the genome of *E. coli* SP15 as a matrix and the tags were amplified from the plasmid pET‐30‐Ek/LIC (Novagen) with primers listed in Table S2. The three PCR products were assembled by NEB Hifi cloning and then digested by *Eco*RI and *Sal*I for cloning in pK184. The other plasmids were built from pAGO‐15, after its linearization and the exchange of the *hlyF* ORF with the different alleles of *cprA* from *P. aeruginosa* or the different CprA orthologs. The plasmid pAGO‐15 was modified with the QuikChange site‐directed mutagenesis method (Agilent Technologies) to obtain pAGO‐16 harboring *hlyF* mutated at Y163F and K167A.

The expression plasmids were built from pJN105 to obtain the genes under the control of the P*ara*BAD promoter. The N‐terminally tagged *hlyF* and *cprA* were amplified by PCR from pAGO‐15 and pAGO‐32 with the primers listed in Table S2, digested with *Sac*I and *Pst*I and ligated in pJN105 digested with the same enzymes.

### 2.3 Mutants and complemented *P. aeruginosa* strains construction

The deletion mutants of *P. aeruginosa* PAKΔ*cprA*, PA14Δ*cprA* and PA14Δ*pmrAB* were constructed by allelic exchange (Schweizer, 1992) as described by Bolard et al. (2019). Briefly, the region upstream and downstream of the target gene was PCR amplified using primers cprAupFor/cprAupRev and cprAdownFor/cprAdownRev, respectively, for PAKΔ*cprA* and PA14Δ*cprA* and primers PCR‐ipmrABC1/C2 and PCR‐ipmrABC3/C4, for PA14Δ*pmrAB* (Table S2). For both mutants, the PCR products were either cloned into the pKNG101 suicide vector (Kaniga et al., 1991) by one‐step sequence and ligation‐independent cloning (SLIC) (Jeong et al., 2012) for PAKΔ*cprA* or cloned into the pCR2.1‐TOPO vector (ThermoFisher) and subcloned in pKNG101 by restriction ligation with *Bam*HI/*Apa*I for PA14Δ*pmrAB*. The resulting plasmids pKNGΔ*cprA* and pKNGΔ*pmrAB* maintained in *E. coli* CC118λ*pir* and *E. coli* HB101, respectively, were then mobilized into PAK or PA14 by triparental mating as previously described (Kaniga et al., 1991). Transconjugants in which the double recombination events occurred, were analyzed by PCR analysis to confirm the gene deletion (Berni et al., 2019). The resulting mutant PA14Δ*pmrAB* was further transcomplemented with plasmid pME6012 and its derivatives (Bolard et al., 2019; Choi & Schweizer, 2006). The cis‐complementation of PAK∆*cprA*, PA14∆*cprA* and the construction of the overproducing strain PAO1 attB::*PRha*‐*cprA* was performed by the insertion of *cprA* under a rhamnose inducible promoter in the *attB* chromosomic site. The *cprA* gene and His‐ and S‐tags were amplified by PCR from the matrix pAGO‐32 using cprARhaup and cprARhadown and cloned by SLIC into the miniCTX1‐rhaSR‐*Prha*BAD (Meisner & Goldberg, 2016). Transfer of MiniCTX‐*PRha*‐*cprA* in *P. aeruginosa* PAKΔ*cprA* and PA14Δ*cprA* strains was carried out by triparental mating and then was selected for the insertion of *PRha*‐*cprA* in the *attB* site.

### 2.4 Purification of bacterial OMVs

The detailed protocol is described in the Figure S1. Briefly, after a 8 h culture, the supernatant was recovered after centrifugation, sterilized by filtration, concentrated through a 100‐kDa MWCO tangential flow filtration unit and then ultracentrifuged. Residual and surface proteins from the sample were digested with the Pronase and OMVs were washed out by ultracentrifugation before undergoing an iodixanol density gradient ultracentrifugation, allowing the selection of fractions containing pure OMVs. The concentration of OMVs in the suspension was correlated with the protein concentration measured by BCA protein assay.

### 2.5 Preparation of liposomes from OMVs lipids

Lipids from *P. aeruginosa* OMVs were extracted using a method adapted from Bligh and Dyer (1959), with the solvent ratio 1:1:0.8 MeOH::CH_2_Cl_2_:H_2_O. This protocol has been described to recover only lipids, without protein or LPS contaminants, the latter being retained in the aqueous phase (Katz et al., 1999). Following the drying of the organic phase under N_2_, the lipid pellets were resuspended in DMEM 1X without phenol red (ref 21063, Gibco). Well‐calibrated—liposomes were then formed by lipid extrusion through 200 nm and 100 nm membranes, according to the manufacturer's instruction about the Avanti Mini Extruder Extrusion Technique (Avanti Polar Lipids Inc). Lipid concentrations in the liposome solution were quantified by the sulpho‐phospho‐vanillin (SPV) lipid assay, following the method of Izard and Limberger (Izard & Limberger, 2003) with Triolein as reference (ASTM Triolein Solution, ref 44896‐U, SigmaAldrich).

### 2.6 Eukaryotic cell culture

HeLa cells (ATCC CCL‐2), GFP‐LC3 HeLa cells and HeLa‐Difluo hLC3 cells (Invivogen) were cultured as previously described (David et al., 2022). THP‐1 cells and associated genetically invalidated cells (Invivogen, THP1‐Null2, thp‐kocasp4z, thp‐konlrp3, thp‐kogsdmdz) were cultured and maintained in RPMI supplemented with 10% heat‐inactivated FCS at 37°C, 5% CO_2_. THP‐1 cells were first pre‐stimulated with 10 ng/mL of IFNγ (Invivogen, rcyec‐hifng) overnight and subsequently primed with 1 µg/mL of LPS (Invivogen, tlrl‐eklps) for 3 h. Cells were washed three times in PBS and then seeded at the density of 10^6^ cells/mL in 24 well plates. Different amounts of specified OMVs were then incubated with cells for 18 h before analysis.

### 2.7 Cell lysis and IL‐1β release determination

Cell culture supernatants were harvested and centrifuged at 2000 × *g* for 10 min to remove cellular debris. LDH release, a marker of cell lysis, was assayed in 50 µL of the supernatant by using the Pierce kit (ThermoFisher, 88953) according to the manufacturer's instructions. IL‐1β release was assayed by ELISA on 100 µL of the supernatant, beforehand diluted by 2, by using the Invitrogen kit (88‐7261).

### 2.8 Transmission electron microscopy (TEM)

Negative staining of OMVs for TEM was performed according to standard procedures. Briefly, 5 µL of OMV samples, with equivalent volume/bacteria OD_600nm,_ were added to carbon‐coated copper mesh grids and stained with 1% uranyl acetate for 1 min. The grids were examined with a Jeol JEM‐1400 (JEOL Inc, Peabody, MA, USA) at 80 kV. Images were acquired using a digital camera (Gatan Orius, Gatan Inc, Pleasanton, CA, USA).

### 2.9 Cryoelectron microscopy (Cryo‐EM)

Isolated OMVs were visualized by cryoelectron microscopy. Briefly, 3 µL of the sample were deposited onto glow‐discharged lacey carbon grids and placed in the thermostatic chamber of a Leica EM‐GP automatic plunge freezer, set at 20°C and 95% humidity. The excess solution was removed by blotting with Whatman n°1 filter paper for 2.5 s and the grids were immediately flash‐frozen in liquid ethane at −185°C. Images were acquired on a Talos Arctica (Thermo Fisher Scientific) operated at 200 kV in parallel beam condition with a K3 Summit direct electron detector and a BioQuantum energy filter (Gatan Inc.). Energy‐filtered (20 eV slit width) image series were acquired with Digital Micrograph software at a pixel size of 0.85Å between −1 and −1.5 µm defocus.

### 2.10 Dynamic light scattering (DLS)

OMVs were analyzed by DLS using a Zetasizer Nano ZS (Malvern Instruments Ltd.) operating at a temperature of 25°C. The OMVs were diluted 10‐fold in PBS for measurements in triplicates and analyzed using a spectrophotometer MACRO cuvette in crystal PS (ref BSA001, Biosigma S.p.A).

### 2.11 Mouse model

Male, 8‐week‐old, C57Bl/6J mice (ENVIGO, France) were infected intraperitonally with 2.10^7^ CFU. Bacteria were cultivated in LB overnight and then 8 h in M63 minimal medium (see above) with 0.1 mM MgCl_2_ for the wild‐type (WT) strain and the mutant strain and with 0.1 mM MgCl_2_ and 6 mM L‐rhamnose (Sigma Aldrich) for the complemented strain. The severity of the clinical signs was evaluated blindly by scoring (Table S3). Any animal presenting at least one clinical sign with a score of 3 led to humanely sacrifice the animal in accordance with decree N°2013‐18 of 1 February 2013. All the experimental procedures were carried out in accordance with the European directives for the care and use of animals for research purposes and were validated by the local and national ethics committees. Protocol number 2023040715164643. 8 h post‐infection, tissue proteins were extracted from the spleen with RIPA (0.5% deoxycholic acid, 0.1% sodium dodecyl sulfate, 1% Igepal in Tris‐buffered saline pH = 7.4) as previously described (Chagneau et al., 2023). Clear lysates were processed for ELISA using commercial kits (Duoset R&D Systems, Lille, France) for Interleukin‐1β (IL‐1β). Data are expressed as picograms of cytokines per milligram of tissue protein.

### 2.12 Bioinformatics analysis

The tridimensional structure predictions of HlyF and CprA proteins from *E. coli* and *P. aeruginosa* were obtained using Alphafold 2 (Galaxy Version 2.3.1**+**galaxy2). The proteins were visualized using ChimeraX 1.3. The protein structure comparison was performed using the iPBA web server (Gelly et al., 2011). For the phylogenetic tree of *P. aeruginosa*, we constructed a Randomized Axelerated Maximum Likelihood tree (RAxML version 8.2.11) from a Mafft alignment of 1000 CDS extracted from each genome (www.bv‐brc.org). The tree has been displayed and annotated with the online tool iTol (itol.embl.de/).

To analyze the allele distribution amongst *P. aeruginosa* isolates, the nucleotide sequences of *cprA* from the strains present in the phylogenetic tree were retrieved from the National Center for Biotechnology Information (NCBI). Then, the amino acid sequence corresponding to each allele was blasted against the *P. aeruginosa* database on the PubMLST.org website (Jolley et al., 2018) and from the National Reference Center for Antibiotic Resistance of *P. aeruginosa* (NRC‐RA Besançon, France).

To analyze the presence of CprA orthologs in other bacterial core genomes a pangenome analysis of each species was performed using reliable genomes from the NCBI genome database. Genome sequences of each species were obtained from the GenBank database and selected for analysis according to the following criteria: (i) keeping full strain genome representation instead of partial, (ii) excluding strains with assembly anomaly such as chimeric, contaminated, misassembled and (iii) filtering out strains with abnormal genome length, low‐quality sequences, untrustworthy as types, unverified source organisms, many frameshifted proteins, abnormal gene to sequence ratio. The 3371 genomes selected, were annotated using prokka (Seemann, 2014) and are submitted to a pangenome analysis using roary (with options: ‐cd 100 ‐i 80) for each bacterial species (Page et al., 2015). In this analysis, ‘core genes’ are defined as those conserved in all isolates.

### 2.13 Statistical analysis

All statistical analyses were performed with Prism 10.1.0 software. Data are represented as the mean ± SD. Significance was determined by a one‐way ANOVA (analysis of variance) for IL‐1β measurement in mice spleen, by a two‐way ANOVA followed with Tuckeys’ multiple comparisons testing for IL‐1β secretion, cell death in human monocytes and for mice clinical score and by a Mantel–Cox test for mice survival. ****p < 0.0001, ***p < 0.001, **p < 0.005, *p < 0.05, ns: not significant, *p* > 0.05.

## 3 RESULTS

### 3.1 CprA exhibits structural homology with HlyF

**FIGURE 1:**
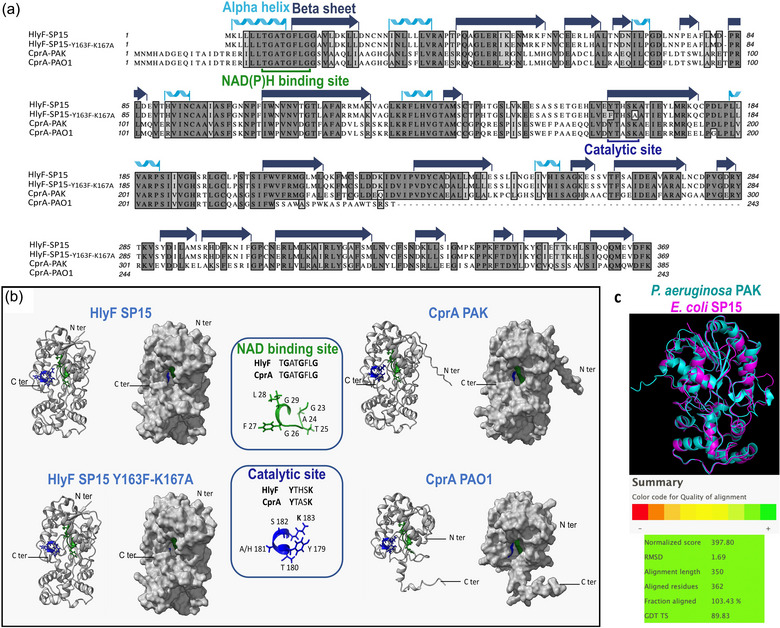
Amino acid homology and structural prediction of CprA and HlyF. (a) Multiple sequence alignment of HlyF and CprA orthologous proteins from *E. coli* strain SP15 and *P. aeruginosa* strains PAK and PAO1 was performed using Clustal Omega. The mutant of HlyF was constructed by site‐directed mutagenesis of the predicted catalytic site leading to the two substitutions Y163F and K167A. The CprA protein from *P. aeruginosa* strain PAO1 is truncated due to a deletion of the cytosine at position 670 of the gene. This results in a frameshift and a premature stop codon at position 245. The residues' identity and similarity are highlighted in dark grey and light grey, respectively. (b) Alphafold 2 protein structure prediction was used to show the three‐dimensional structure of the HlyF and CprA proteins from *E. coli* and *P. aeruginosa*. In both panels, the NAD(P)H binding sites and catalytic sites are depicted in green and blue, respectively. (c) The iPBA web server was used to compare the secondary structure of CprA (in blue) and HlyF (in pink). (https://www.dsimb.inserm.fr/dsimb_tools/ipba/index.php). HlyF, hemolysin F, CprA, cationic peptide resistance A.

CprA has been described in two forms: a full‐length allele found in the PAK strain and a truncated allele found in PAO1 due to an indel mutation that creates a premature stop codon at position 245 (Gutu et al., 2015). CprA from PAK shares 50.13% amino acid residues identity with HlyF and is predicted to belong to the SDR superfamily. The protein features a highly conserved catalytic site and a NAD(P)H binding site as shown in Figure 1a. In PAO1, the truncated form of CprA retains both the catalytic and NAD(P)H binding sites but has lost one alpha helice and eleven beta sheets, which constitute the lower portion of the protein, resulting in a change in its conformation. Notably, HlyF and CprA exhibit similar predicted three‐dimensional structures and share a comparable catalytic pocket with the NAD(P)H cofactor binding site located next to the catalytic site (Figure 1b and 1c). The predicted structural similarity between HlyF and full‐length CprA prompted us to investigate whether CprA shares similar properties with HlyF, such as the ability to produce OMVs that modulate autophagy in eukaryotic cells.

### 3.2 When produced in *E. coli*, the function of CprA is similar to that of HlyF

**FIGURE 2:**
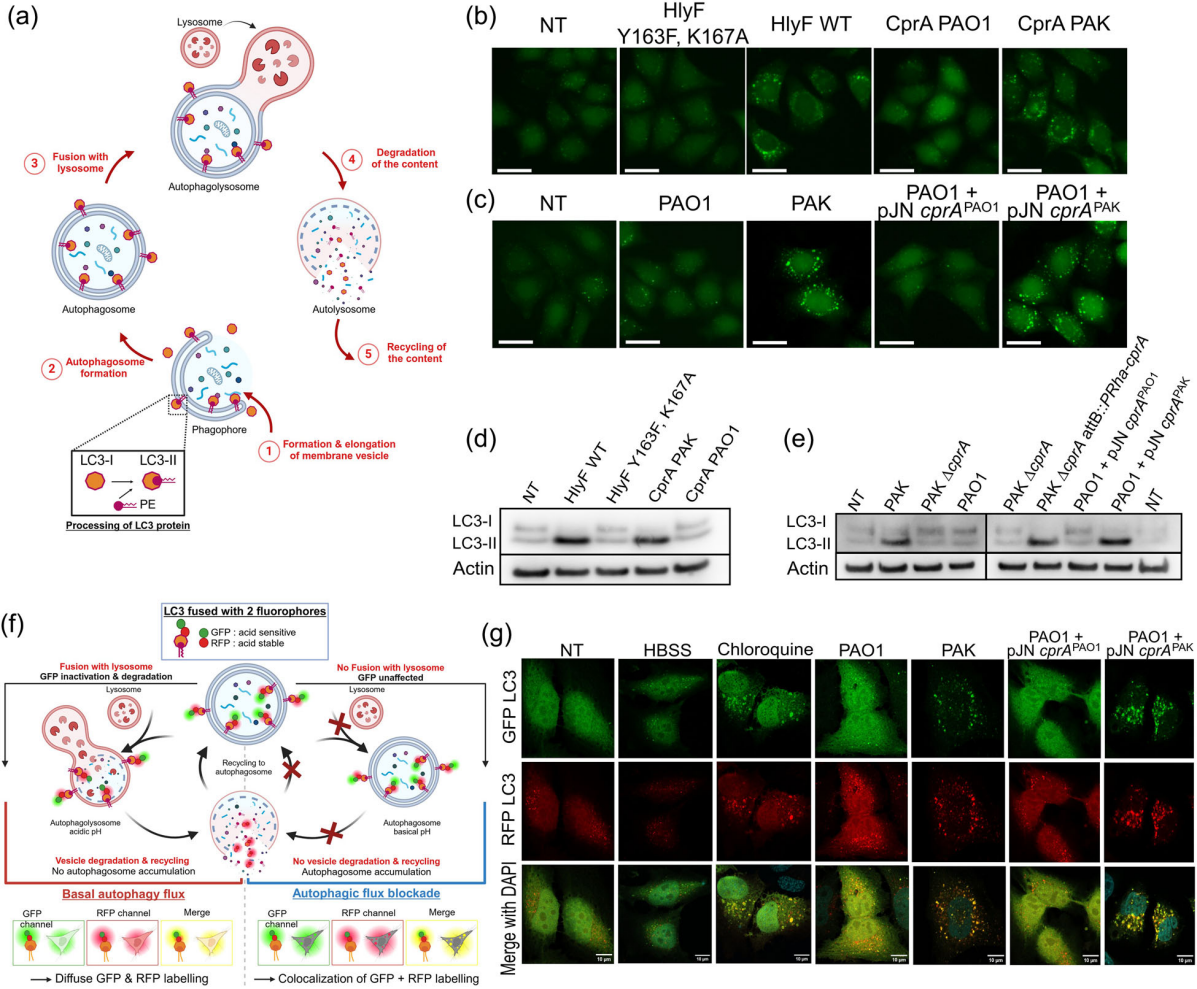
OMVs derived from CprA or HlyF‐producing *E. coli* and OMVs from *P. aeruginosa* that produce a full‐length CprA trigger autophagosomes in HeLa cells and inhibit the autophagy flux of HeLa cells. (a) This basal autophagy flux model describes the following five stages: (1) elongation and formation of a double‐membrane LC3‐II‐dependent vesicle containing cytoplasmic components; (2) newly formed autophagosome; (3) fusion of the autophagosome with the lysosome; (4) degradation and (5) recycling of autophagolysosome contents. Created with BioRender.com. (b, c) Fluorescent microscopy images of GFP‐LC3 (green) in HeLa cells expressing green fluorescent protein–fused with LC3. For panel b, cells were treated for 3 h with 5 µg/mL of OMVs purified from *E. coli* BL21 DE3 strain expressing the WT *hlyF* (*hlyF* WT) from *E. coli* strain SP15 (pAGO‐15), the mutant in the catalytic site (*hlyF* Y163F, K167A) (pAGO‐16) or *cprA* from *P. aeruginosa strain* PAK (pAGO‐32) or strain PAO1 (pAGO‐30). For panel c, cells treated for 5 h with 50 µg/mL of OMVs purified from *P. aeruginosa* strains PAK, PAO1 and PAO1 + pJN cprA^PAK^ (pAGO‐38) or PAO1+ pJN cprA^PAO1^(pAGO‐37). In b and c, the scale bar in the images represents 25 µm. These images are representative of three independent experiments. (d, e) Western blot analysis of LC3 (forms I and II, as autophagy marker) and actin (as loading control) in HeLa cells. For panel d, cells were treated as in panel b. For panel e, cells were treated for 5 h with 50 µg/mL of OMVs purified from *P. aeruginosa* strains PAK, PAK ∆*cprA*, PAO1 and PAO1 + pJN *cprA* ^PAO1^ (pAGO‐37) or for 1.5 h with 50 µg/mL of OMVs purified from *P. aeruginosa* strains PAK ∆*cprA* attB::*PRha*‐*cprA* and PAO1 + pJN *cprA* ^PAK^ (pAGO‐38). Each western‐blot is representative of three independent experiments. In all panels, NT were used as a control. (f) Schema for visualizing basal or blocked autophagy flux in the HeLa‐Difluo hLC3 cell line. The cells express a LC3 protein fused with RFP, which is resistant to acidic conditions and GFP, which is sensitive to acidity. In the case of basal autophagic flux, the GFP‐LC3 signal decreases due to the fusion of autophagosomes with acidic lysosomes. Subsequently, the autophagolysosome undergoes degradation and recycling, preventing the accumulation of autophagosomes. Basal autophagy is visualized through fluorescence microscopy as diffuse GFP and RFP labeling. When the autophagic flux is blocked at the lysosomal fusion step, autophagosomes accumulate with a basic pH, which is visualized as the co‐localization of GFP and/or RFP signals in foci. If the autophagic flux is blocked after the lysosomal fusion step, the autophagolysosome accumulates with an acidic pH, which is observed by a low GFP‐LC3 signal and intact RFP‐LC3 foci (Kimura et al., 2007; Loos et al., 2014). Created with BioRender.com (g) Confocal images of DiFluo HeLa cells were captured under different conditions: NT, deprived by incubation for 5 h in HBSS, treated for 5 h with 50 µM chloroquine or treated with 50 µg/mL of OMVs from *P. aeruginosa* strains PAK, PAO1 and PAO1 + pJN *cprA* ^PAK^ (pAGO‐38) and PAO1 + pJN *cprA* ^PAO1^ (pAGO‐37). NT were used as a control. The scale bar in the images represents 10 µm. These images are representative of three independent experiments. GFP, green fluorescent protein; NT, non‐treated cells; OMV, outer membrane vesicles; PE, phosphoethanolamine; RFP, red fluorescent protein; WT, wild‐type.

To determine whether structural homology leads to conserved function, we have cloned both the truncated (from PAO1) and full‐length (from PAK) variants of *cprA* and expressed them in *E. coli*. We then purified OMVs from *E. coli* expressing these variants and tested their ability to modulate autophagy in HeLa cells, by assessing the accumulation of autophagosomes (Figure 2) (David et al., 2022). The marker of autophagosome LC3 was assessed by immunofluorescence using a fusion LC3‐GFP or analyzed by western blotting to distinguish between the LC3‐I (free) and the LC3‐II (associated with autophagosomes) forms (Figure 2a). Treatment with OMVs derived from *E. coli* harboring the full‐length CprA from the PAK strain resulted in the presence of LC3‐GFP foci (Figure 2b), along with an accumulation of LC3‐II protein, as evidenced by western blotting (Figure 2d). This phenotype closely resembles that observed in cells treated with OMVs produced by *E. coli* producing HlyF (i.e., the accumulation of autophagosomes, as reported in David et al. (2022)). In contrast, OMVs produced by *E. coli* that produce the truncated form of CprA from PAO1 failed to induce the accumulation of LC3‐II or GFP‐LC3 foci. The observed outcome was comparable to the results seen in untreated cells or cells treated with OMVs produced by *E. coli* that produce an inactive form of HlyF with the two mutations in the catalytic domain Y167F and K164A (Figure 1, Figure 2b and 2d). Therefore, the activity of the full‐length form of CprA is similar to that of HlyF when produced in *E. coli*.

### 3.3 OMVs from CprA‐producing *P. aeruginosa* trigger the accumulation of autophagosomes

To evaluate the ability of *P. aeruginosa* expressing *cprA* to induce autophagosomes accumulation through the production of specific OMVs, we used the WT PAK strain, its *cprA* gene‐deleted mutant, the complemented mutant strain PAK ∆*cprA* attB::*PRha‐cprA* and the PAO1 strain transformed with a plasmid expressing either the full‐length or the truncated form of *cprA*, referred to as *cprA^PAK^* or *cprA^PAO1^*, respectively. OMVs were purified from culture supernatants (Figure S1) and visualized using both TEM and transmission electron cryomicroscopy (Cryo‐TEM) and further characterized by DLS. The PAK strain, with or without full‐length CprA, yielded heterogeneous populations of spherical OMVs with a diameter of 140–150 nm ± 50 nm (as observed in panel DLS of Figure S1), which exhibited a single lipid bilayer, confirming their classification as *bona fide* OMVs, as previously reported in the literature (Couto et al., 2015; Zavan et al., 2023). Upon treatment with OMVs isolated from *P. aeruginosa* producing full‐length CprA, cells showed an accumulation of GFP‐LC3 foci (Figure 2c), which is consistent with the increase in LC3‐II compared to the untreated sample (Figure 2e). In contrast, cells treated with OMVs derived from *P. aeruginosa* producing the truncated form of CprA did not show GFP‐LC3 foci or elevated levels of LC3‐II. Thus, full‐length CprA but not its truncated variant, allows *E. coli* and *P. aeruginosa* to produce OMVs that induce accumulation of autophagosomes in eukaryotic cells.

### 3.4 *Pseudomonas aeruginosa* producing CprA produce OMVs that impede the autophagic flux

Autophagy is a dynamic process that involves the formation and degradation of autophagosomes. Previous research has shown that OMVs produced by *E. coli* producing HlyF impede the autophagic flux at the lysosome‐autophagosome fusion stage, leading to the accumulation of autophagosomes (David et al., 2022). To determine whether OMVs derived from *P. aeruginosa* producing a full‐length CprA can impede the autophagic flux at the lysosome‐autophagosome fusion stage, we used HeLa‐Difluo hLC3 cells to monitor the acidification of autophagosomes (Figure 2f). In cells treated with OMVs from *P. aeruginosa* producing a full‐length form of CprA (PAK or PAO1 pJN *cprA* ^PAK^), we observed a pronounced colocalization of GFP and RFP‐positive puncta, similar to the positive control where cells were treated with chloroquine inhibiting the lysosomal fusion (Figure 2g). In contrast, HeLa‐Difluo hLC3 cells treated with OMVs from *P. aeruginosa* producing the truncated CprA (PAO1 or PAO1 pJN *cprA* ^PAO1^), showed a diffuse GFP and RFP labelling with no colocalization, similar to that observed in cells deprived by incubation in HBSS, known to induce a fully functional autophagic flux (Figure 2g). Previous work has demonstrated that the absence of acidification of autophagosomes in cells treated with OMVs from *E. coli* producing HlyF is due to a fusion defect with the lysosome (David et al., 2022). Therefore, our results strongly suggest that OMVs from *P. aeruginosa* producing full‐length CprA, but not the truncated variant, impede the autophagic flux by preventing the fusion between the autophagosomes and the lysosomes.

### 3.5 The production of CprA by *P. aeruginosa* results in OMVs with a greater capacity to activate the non‐canonical inflammasome pathway NLRP3

**FIGURE 3:**
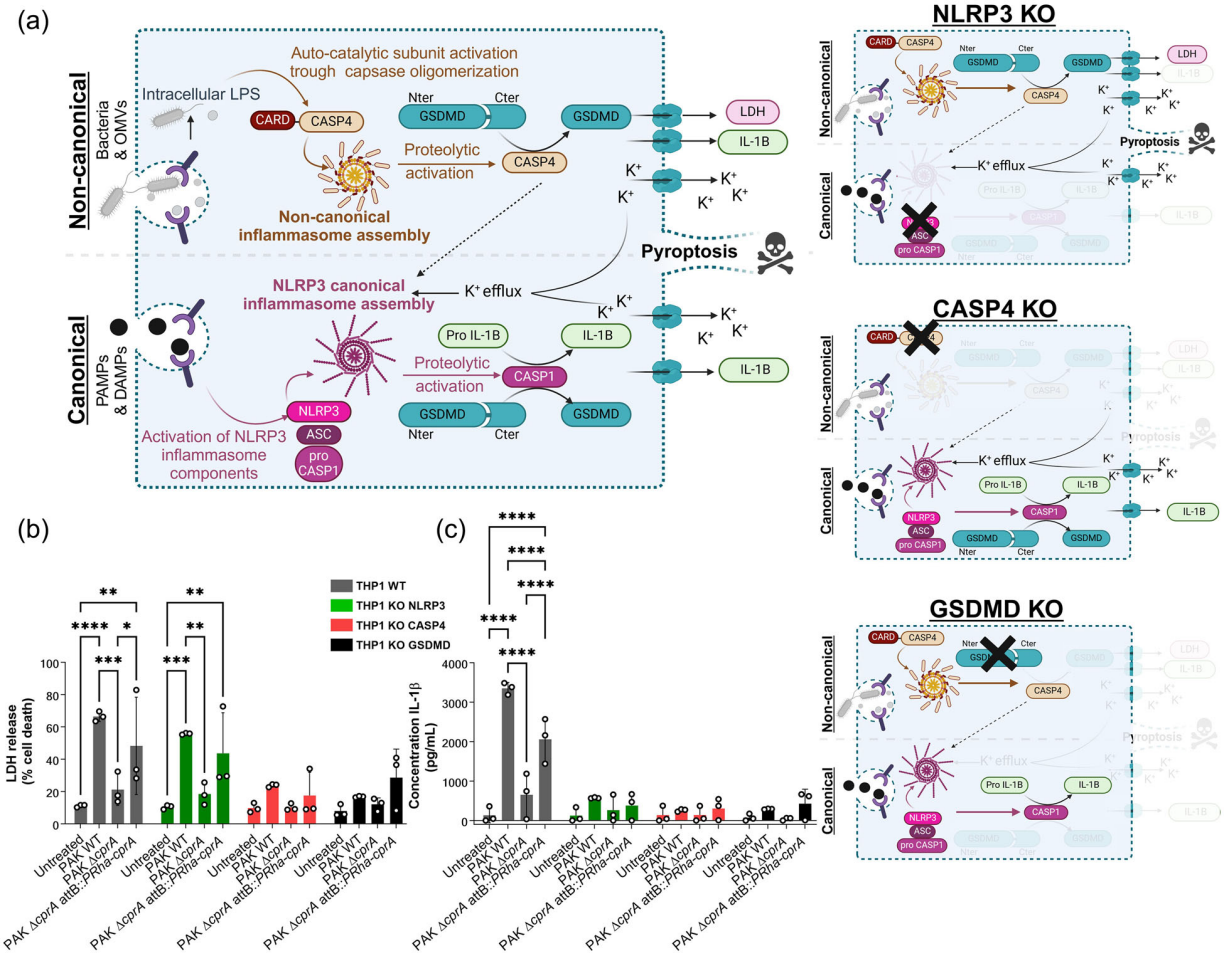
OMVs from *P. aeruginosa* producing the full‐length CprA enhance IL‐1β secretion and cell death in human monocytes. (a) Schema of the simplified canonical pathway of NLRP3 inflammasome and non‐canonical inflammasome after activation with pathogen or danger‐associated patterns (PAMPs or DAMPs), Gram‐negative bacteria or OMVs. The assembly of the NLRP3 canonical inflammasome relies on the sensing of cytoplasmic stress signals such as K+ efflux, in response to prior detection of extracellular PAMP and DAMPs and leads to the activation of CASP1. Assembly of the non‐canonical inflammasome occurs after sensing of cytoplasmic LPS (from bacteria or OMVs) by the CARD domain of CASP4, leading to the activation of its own autocatalytic subunit. Pro‐IL‐1β was cleaved to its mature form (IL‐1β) by the activated CASP1. Activated CASP1 and CASP4 cleave gasdermin‐D (GSDMD), leading to pore formation at the plasma membrane, which triggers cell death by pyroptosis and release of the cytosolic contents such as of LDH, IL‐1β and K^+^, acting as a stress signal to the surrounding cells. K^+^ efflux induces the assembly of the NLRP3 canonical inflammasome and the maturation of pro‐IL‐1β via CASP1 (Li et al., 2021; Zito et al., 2020; Man & Kanneganti, 2015). Here, we used human monocytic THP‐1 WT cells or THP‐1 cells knocked out for NRLP3, CASP4 or GSDMD. In NLRP3 KO, there is no more secretion of mature IL‐1β, but LDH secretion, through CASP4‐mediated pyroptosis, still occurs. In CASP4 KO and in GSDMD KO, the cell death is abrogated and the mature IL‐1β is severely impaired when the non‐canonical inflammasome is activated by OMVs. Created with BioRender.com. (b) Release of LDH and (c) IL1‐β from primed WT, NLRP3 KO, CASP4 KO THP‐1 and GSDMD KO THP‐1 cells treated overnight with 12.5 µg/mL of OMVs from *P. aeruginosa* strains PAK, PAK ∆*cprA* and PAK ∆*cprA* attB::*PRha*‐*cprA*. For b and c, the graphs show the mean and the standard deviation of three independent experiments for each condition, each point represents the value obtained in one experiment. Significance was determined by a 2‐way ANOVA, with Tuckey's multiple comparisons test. ****p < 0.0001, **p < 0.005, *p < 0.05. CASP 1, caspase‐1; CASP 4, caspase‐4; OMV, outer membrane vesicles; WT, wild‐type.

The non‐canonical inflammasome pathway is induced by the activation of the NLRP3 inflammasome by cytosolic lipopolysaccharide (LPS). Previous research has shown that OMVs from bacteria producing HlyF were more prone to activate the non‐canonical inflammasome pathway than OMVs purified from bacteria producing an inactive form of HlyF (David et al., 2022). It was hypothesized that the expression of *cprA* could affect the ability of OMVs to activate the non‐canonical pathway of NLRP3 inflammasome. Human monocytic THP‐1 WT cells were used along with THP‐1 cells knocked out for NRLP3 (THP1^NRLP3KO^), an intracellular sensor that is part of the caspase‐1 activating complex, or for Caspase‐4 (THP1^CASP4KO^) or Gasdermin‐D (THP1^GSDMDKO^), two essential components of the non‐canonical inflammasome pathway (Figure 3a). The cells were exposed to OMVs from *P. aeruginosa* PAK WT, the mutant PAK ∆*cprA* and the complemented strain (PAK ∆*cprA attB::PRha*‐*cprA*). Although the effect was smaller with the OMVs from the mutant PAK ∆*cprA*, OMVs from the 3 strains induced THP‐1 cell lysis (measured by LDH release) and IL‐1β release (measured by ELISA) in a Caspase‐4 and GSDMD‐dependent manner. However, no cell death was observed in an NLRP3‐dependent manner. These results indicate that *P. aeruginosa* OMVs activate the non‐canonical inflammasome pathway (Figure 3b and 3c). Exposure of cells to PAK WT and PAK ∆*cprA* *attB::PRha‐cprA* OMVs resulted in a significant increase in cell lysis and IL‐1β release compared to PAK ∆*cprA*. This process was found to be dependent on Caspase‐4 and GSDMD, as shown in Figure 3. Our findings indicate that OMVs from *P. aeruginosa* which produce CprA, are much more prone to activate the non‐canonical inflammasome pathway. This effect could be explained by the blockage of the autophagic flux, which inhibits the primary negative feedback mechanism of non‐canonical inflammasome activation, as observed with OMVs from HlyF‐producing *E. coli* (David et al., 2022).

### 3.6 Role of the PmrAB two‐component system in CprA‐mediated OMVs production and autophagic disruption

**FIGURE 4:**
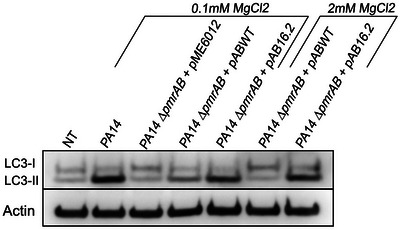
CprA activity is dependent on the two‐component system PmrA/B. Western blot analysis of LC3 and actin in HeLa cells treated for 1 h with 50 µg/mL of OMVs from *P. aeruginosa* strain PA14, the ∆*pmrAB* mutant carrying the empty vector (pME6012), the mutant strain complemented with a native *pmrAB* allele (pABWT) or complemented with a gain‐of‐function *pmrAB* allele (pAB16.2). Bacteria were cultivated in M63 supplemented with two different concentrations of MgCl_2_: one (0.1 mM) described to activate the PmrAB two‐component regulatory system (Mcphee et al., 2006) and one (2 mM) described to inactivate the PmrAB two‐component regulatory system. NT was used as a control. Blots are representatives of three independent experiments. NT, non‐treated cells; OMV, outer membrane vesicles.

Previous studies have shown that the PmrAB two‐component system plays a regulatory role in the transcriptional activation of *cprA*, but the role and enzymatic activity of CprA in the bacteria was unclear at the time (Bolard et al., 2019; Mcphee et al., 2006). Given the functional similarities between the CprA allele of PA14 and PAK (Figure S3a,b,c), we chose to evaluate the role of PmrAB in CprA‐mediated OMVs production in PA14, a more virulent strain that causes disease in a wide range of organisms. To test whether PmrAB boosts the toxicity of OMVs, we performed a comparative analysis of the effects of OMVs obtained from the WT *P. aeruginosa* PA14 strain, its *pmrAB*‐deficient isogenic mutant harboring either the empty vector (PA14 ∆*pmrAB* + pME6012) or the vector encoding the WT *pmrAB* allele (PA14 ∆*pmrAB* + pABWT) (Bolard et al., 2019). As PmrAB is known to be activated by a low concentration of Mg^2+^, we cultivated the strains in a medium with a low concentration of MgCl_2_ (0.1 mM) (Mcphee et al., 2006). Western blot analysis showed an accumulation of LC3‐II protein levels in cells exposed to OMVs from the WT PA14. In contrast, the strain that lacked the PmrAB did not show any accumulation of LC3‐II, similar to the untreated cells. Complementation with the WT *pmrAB* allele partially restored the ability of the mutant strain to produce OMVs that induce LC3‐II accumulation (Figure 4). These findings highlight the crucial role of a functional PmrAB two‐component system in producing OMVs that impede the autophagic flux when environmental conditions activate the two‐component system.

### 3.7 PmrB gain‐of‐function mutations increase the production of OMVs that block autophagic flux

Exposure to colistin may select for *pmrB* mutants that confer a gain of polymyxin resistance, as observed in clinical isolates (Bolard et al., 2019; Moskowitz et al., 2012; Moskowitz et al., 2004). This phenomenon is attributed to the elevated mutation rate that occurs in this locus upon colistin selection (Kapel et al., 2022). These mutations are referred to as ‘gain‐of‐function’ mutations because they activate the PmrAB regulon in the absence of environmental signals (Moskowitz et al., 2004). To investigate whether a *pmrAB* allele described as a gain‐of‐function allele could also induce the production of OMVs that block the autophagic flux, we treated HeLa cells with OMVs from PA14 Δ*pmrAB* complemented with either the WT *pmrAB* allele (pABWT) or with the *pmrAB* harboring the deletion ∆L172 in PmrB (pAB16.2), identified as a *pmrAB* gain‐of‐function allele (Bolard et al., 2019). OMVs were produced by cultivating bacteria in the minimum medium M63 supplemented with either an activating (0.1 mM) or an inhibiting (2 mM) concentration of MgCl_2_. We observed a clear accumulation of the LC3‐II protein in HeLa cells treated with OMVs obtained from the strain complemented with the *pmrAB* gain‐of‐function allele (i.e., magnesium non‐responsive allele), regardless of the concentration of MgCl_2_ (Figure 4). In contrast, OMVs from the strain complemented with the WT *pmrAB* allele (i.e., magnesium‐responsive allele) blocked the autophagy flux only when the bacteria are cultivated in a MgCl_2_ concentration activating the PmrAB two‐component system. The toxicity of OMVs on cells was abolished when the bacteria were cultivated with a high concentration of MgCl_2_, inactivating PmrAB. This result shows that the *pmrAB* gain‐of‐function allele enables the constitutive production of toxic OMVs, regardless of environmental factors.

### 3.8 CprA is a virulence factor of *P. aeruginosa* in a mouse sepsis model

**FIGURE 5:**
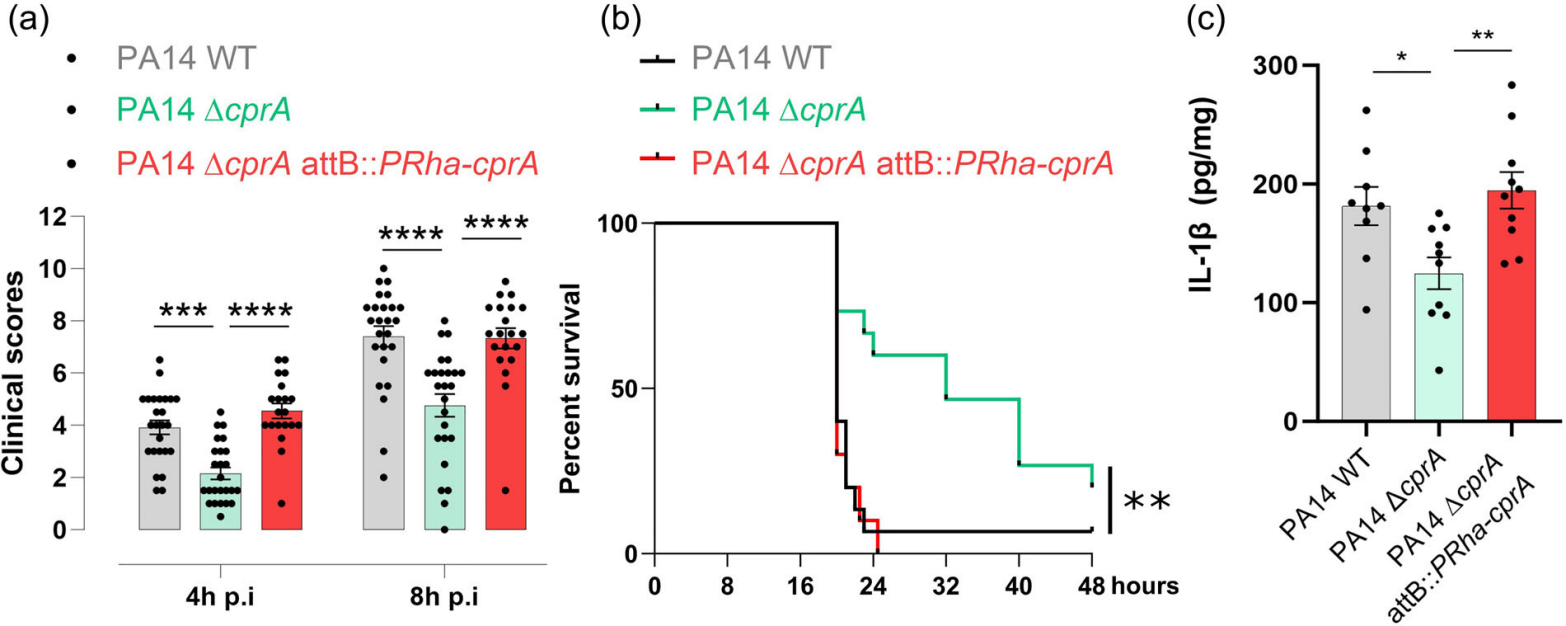
CprA increases pathogenicity during sepsis. C57BL/6 mice were infected intraperitoneally with WT *P. aeruginosa* PA14 strain, *cprA* isogenic mutant PA14 Δ*cprA* or with PA14 ∆*cprA* strain complemented with *cprA* (PA14 ∆*cprA* attB::*PRha*‐*cprA*). (a) Clinical score according to Table S3 at 4 and 8 hours post‐injection. The results shown are pooled from three independent experiments (*n* = 20–25). Differences between the experimental groups were evaluated by two‐way ANOVA followed by Tuckey's results: *p < 0.05; **p < 0.01; ***p < 0.001; ****p < 0.0001. Mean values ± SEM are shown. (b) The time to humane euthanasia (when the clinical score reached a predefined threshold) was monitored to build the survival curve. Results are pooled from two independent experiments and the total number of animals is shown (*n* = 10–15). The difference between the experimental groups was evaluated by the log‐rank test (Mantel‐Cox): **p < 0.01. (c) Protein levels of the cytokines IL1‐β from mouse spleen 8 h post‐infection were determined in proteins extracted from tissues using ELISA. The graphs show the mean and the standard deviation from an independent experiment (*n* = 10). Significance was determined by a 1‐way ANOVA, **p < 0.005, *p < 0.05. ANOVA, analysis of variance; SEM, standard error of the mean; WT, wild‐type.

Subsequently, the role of CprA in the virulence of *P. aeruginosa* was evaluated in a mouse model of infection. Mice were intraperitoneally injected with 2.10^7^ CFU of PA14 WT, PA14 ∆*cprA* mutant or the complemented PA14 ∆*cprA attB::PRha*‐*cprA*. The clinical scores for mice infected with the mutant strain were significantly lower than those infected with the WT or complemented strain at both 4 and 8 h post‐infection (Figure 5a, Table S3). Between 20 and 24 h post‐infection, the WT and complemented strains caused mortality rates of 94% and 100%, respectively. In contrast, infection with the PA14 ∆*cprA* mutant strain resulted in a statistically significant delay in survival kinetics, with a median of 32 hours post‐infection (Figure 5b). The lower clinical scores and the related mortality rates observed in the PA14 ∆*cprA* mutant compared to PA14 with *cprA* (WT or complemented strain), may be explained by a lower level of inflammation exerted by a strain lacking *cprA*, as observed in THP‐1 cells (Figure 3). To verify this assessment, the levels of the pro‐inflammatory cytokine IL‐1β induced by the non‐canonical pathway of NLRP3 inflammasome were evaluated in mice spleen 8 h post‐infection. A reduction in the inflammatory response was observed in the spleen of mice infected with the PA14 ∆*cprA* mutant strain in comparison to the WT or complemented strain. This was demonstrated by the observation of reduced levels of interleukin IL‐1β (Figure 5c). These results demonstrate that CprA contributes to the virulence of *P. aeruginosa* and is responsible for a stronger inflammatory response. This is likely responsible for the observed clinical scores and associated survival kinetics.

### 3.9 The *cprA* gene is ubiquitously present in *P. aeruginosa*

Analysis of available genomes in the databases indicated that the *cprA* gene and its genetic environment are part of the core genome of *P. aeruginosa*. This is true for the PAO1, PAK and PA14 strains, as well as the more phylogenetically distant PA7 strain. The genetic organization upstream and downstream of *cprA* includes essential genes, such as those encoding subunits of cytochrome C oxidase which is an enzyme necessary for aerobic respiration and the aerotaxis receptor Aer (Figure S2a). Out of the 5286 genomes obtained from the PubMLST database (2797 genomes) and the collection of the French National Reference Center for Antibiotic Resistance *P. aeruginosa* strains (2489 genomes), only 17 (0.32%) displayed a truncated variant of CprA. Amongst these, 14 had a variant identical to the strain PAO1 (refer to Figure S2c). Twelve major alleles were identified and their distribution was not dependent on the *P. aeruginosa* clades, unlike the well‐known type III secretion system exotoxins ExoU (Clade B) and ExoS (clade A) and the exolysin ExlA (most of the clade C) (Figure S2b,c). The most common allele is found in CprA*^ATCC27853^*, found in 81 % of the isolates (Figure S2c). This allele is present in the most prevalent epidemic high‐risk clones such as ST111, ST175, ST233, ST235, ST244, ST257, ST308, ST357 and ST654 (Del Barrio‐Tofiño et al., 2020). The CprA allele from the outlier strain PA7 is notably the most genetically divergent (Figure S3a and Figure S2c) compared to the other alleles. We assessed the capacity of the most frequent allele, CprA*^ATCC2785^*, the allele with the highest rate of SNPs CprA*^PA7^* and the CprA*^PAK^* and CprA*^PA14^* alleles, which have already been shown to be functional in this study, to produce toxic OMVs. The two *E. coli* hosting the constructs CprA*^ATCC27853^* and CprA*^PA7^* led to the production of OMVs blocking autophagy, just as does CprA*^PA14^* and CprA*^PAK^* (Figure S3b,c). We also confirmed the production of autophagy‐blocking OMVs in clinical strains isolated from patients at the University Hospitals of Toulouse with cystic fibrosis, ear‐nose‐throat infections and infections due to medical devices (Figure S3d). After sequencing the *cprA* gene in these isolates, we identified the CprA*^ATCC27853^* allele in all of them, which is consistent with the prevalence of this allele amongst *P. aeruginosa*. These results show that all *P. aeruginosa* strains, with the exception of a few strains harboring a truncated CprA allele such as PAO1, are potentially capable of producing OMVs that inhibit autophagy.

### 3.10 CprA and HlyF represent a novel family of virulence determinants in Gram‐negative bacteria

**FIGURE 6:**
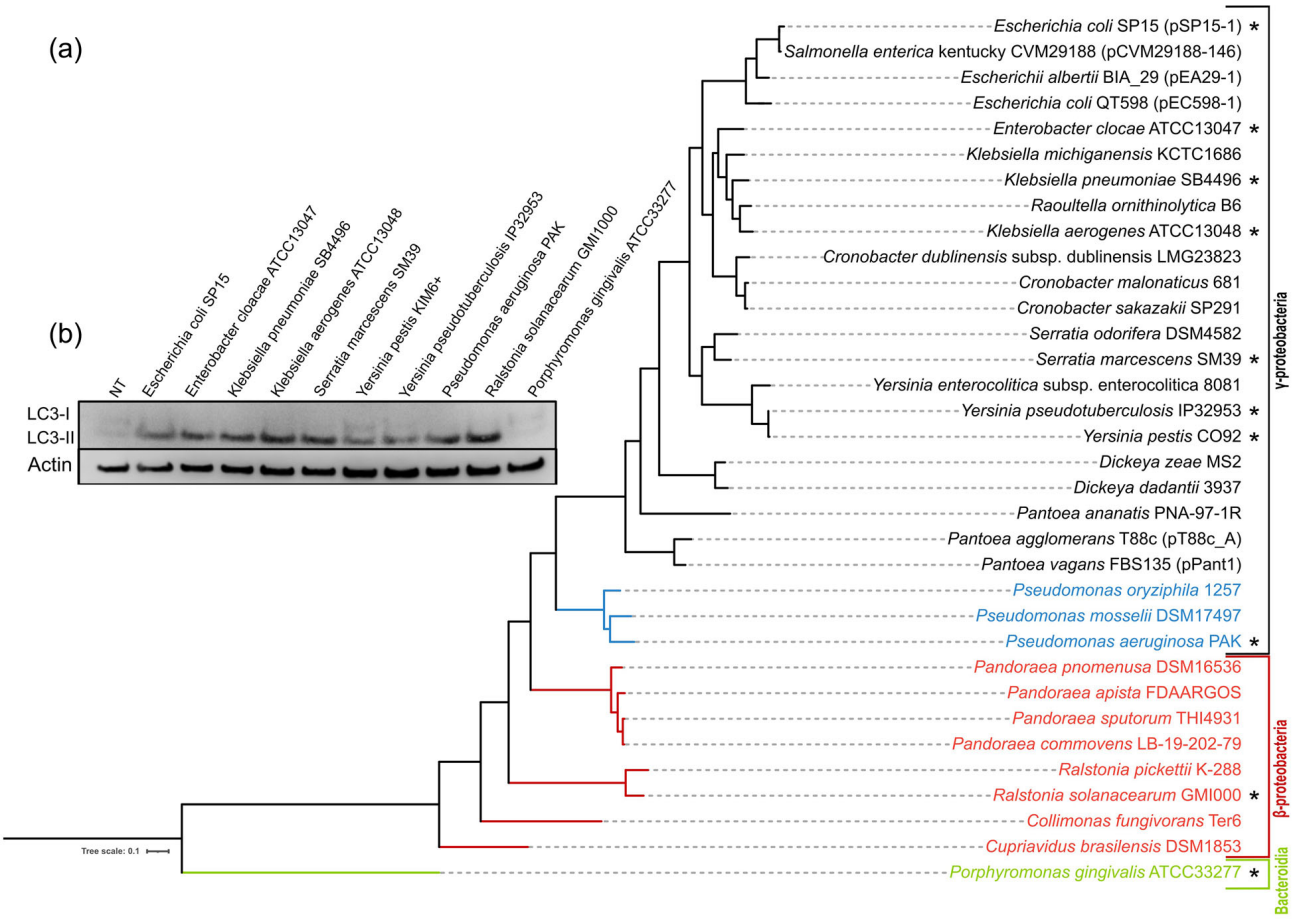
OMVs from *E. coli* expressing various CprA orthologs trigger the accumulation of autophagosomes in HeLa cells. (a) Maximum likelihood phylogenetic tree of HlyF/CprA orthologs present in various bacterial species, rooted on the most distant and non‐functional protein found in *Porphyromonas gingivalis*, here the only representative of the *Bacteroidia* class. This protein shares the amino acids present in the NAD(P)H binding site and in the catalytic site with CprA, but lacks the highly conserved C‐terminus part of the protein with D‐F/Y‐K residues. Other bacterial species belong to two main classes: the *Gammaproteobacteria* and the *Betaproteobacteria*. The colors indicate the taxonomic order: black for the *Enterobacterales*, blue for the *Pseudomonadales* and red for the *Burkholderales*. Orthologs activity from the strains marked with an asterisk (*) are tested in panel b. The scale bar represents the number of substitutions per site. (b) Western blot analysis of LC3 and actin in HeLa cells treated for 3 h with 5 µg of OMVs purified from *E. coli* BL21 (DE3) strain expressing *hlyF* from *E. coli* SP15 (pAGO‐15) or the orthologs from *E. cloacae* ATCC13047 (pAGO‐17), *K. pneumoniae* SB4496 (pAGO‐19), *K. aerogenes* ATCC13048 (pAGO‐21), *S. marcescens* SM39 (pAGO‐23), *P. aeruginosa* PAK (pAGO‐32)*, R. solanacearum* GMI1000 (pAGO‐29), *P. gingivalis* ATCC33277 (pAGO‐42) or with 20 µg of OMVs purified from *E. coli* BL21 (DE3) strain expressing the orthologs from *Y. pestis* KIM6+ (pAGO‐53) and *Y. pseudotuberculosis* IP32953 (pAGO‐55). NT was used as a control. The blot shown is representative of three independent experiments. NT, non‐treated cells; OMV, outer membrane vesicles.

Given the relative taxonomic distance between *E. coli* and *P. aeruginosa*, it was of interest to ascertain whether CprA and HlyF orthologs were found in other bacteria. A number of cryptic SDRs, similar to CprA and HlyF, have been identified in the genome of Gram‐negative bacteria. The potential orthologs exhibit a protein size ranging from 360 to 385 residues and a minimum of 40% amino acid identity, including the key residues of the NAD(P)H binding site (TG.TGF.G) and the catalytic site (YT.SK), which are typical of SDRs. CprA/HlyF orthologs have been found mainly in bacteria belonging to the class of the *Gammaproteobacteria* and especially the orders of *Pseudomodales* and *Enterobacterales*. Orthologs are also found in the *Betaproteobacteria* class, especially in the *Burkholderiales* order (Figure 6a and Figure S4a). The majority of these orthologs are encoded on the bacterial chromosome, with a similar GC% to the rest of the genome, suggesting that they belong to the core genome (Figure S4b). This is particularly true for 18 bacterial species for which several genomes could be used for a pangenome analysis (Table S4). Although the number of core genes varied amongst species, more than 93% of isolates in each species possess an HlyF ortholog, with the exception of *D. dadantii* and *P. agglomerans*, indicating that the genes are highly conserved amongst the genome. The absence of an HlyF ortholog in a minor portion of genomes could be explained by sequencing or assembly errors in retrieved genomes. We also observed a larger amount of *P. agglomerans* missing the HlyF ortholog, certainly because the orthologs are encoded by a large plasmid (110 to 500 kbp), such as those from *E. coli, E. albertii, S. enterica* and *P. vagans* (Figure 6a).

To investigate the roles of these putative SDRs, we purified OMVs produced by *E. coli* expressing different orthologous proteins from pathogens that are of interest in terms of human, mammal and plant health such as *S. marcescens, K. pneumoniae, Y. pestis* and *R. solanacearum* (Figure 6a). Strikingly, we consistently observed an accumulation of LC3‐II protein, similar to the results obtained with OMVs from *E. coli* expressing *hlyF* or *cprA* (Figure 6b). All validated orthologs, including the two most distant forms (HlyF from *E. coli* SP15 and HlyF/CprA ortholog from *R. solanacearum* GMI1000), are functional and maintain three‐dimensional structural similarity and conserved residues (Figure S4c,d,e). Furthermore, all of these orthologs possess a protein domain classified as fatty acyl CoA reductases (FARs) in the National Library of Medicine's conserved domain database (CDD) (Wang et al., 2023). All these orthologs, including HlyF and CprA, are characterized by the FAR‐N, SDRe domain (accession: cd05236), but lack the FAR‐C superfamily domain (C‐terminal domain of FAR, accession: cd0971).

Fatty acyl‐CoA is reduced to fatty alcohols by FARs, which can catalyze both saturated and unsaturated C16 or C18 fatty acids (Cheng & Russell, 2004). The homology of CprA, HlyF and their orthologs to FARs suggests that these SDRs may modify the bacteria's outer membrane composition by targeting lipids.

This leads us to the hypothesis that these SDRs could modify the lipid composition of the bacterial OMVs.

### 3.11 Liposomes made with bacterial lipids extracted from OMVs of CprA‐producing bacteria, inhibit autophagy

**FIGURE 7:**
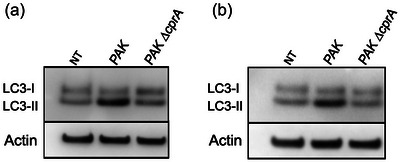
Lipids from CprA producing *P. aeruginosa* trigger autophagosomes accumulation in HeLa cells. Western blot analysis of LC3 and actin in HeLa cells treated (a) for 1 h with 50 µg/mL of OMVs and (b) for 6 hours with an equivalent amount of lipids extracted from 3.33 µg of OMVs from *P. aeruginosa* PAK or PAK ∆*cprA*. In both panels, bacteria were cultivated in M63 supplemented with 0.1 mM MgCl_2_ (described to activate the PmrAB two‐component regulatory system). NT was used as a control. NT, non‐treated cells.

We extracted lipids from the OMVs of *P. aeruginosa* PAK expressing or not *cprA*, using a method adapted from Bligh and Dyer (1959). Following liposome formation through lipid extrusion, HeLa cells were treated with a lipid amount equivalent to the dose of OMVs containing 3.33 µg protein (Figure 7). We observed an accumulation of LC3‐II protein in cells treated with OMVs or liposomes from *P. aeruginosa* PAK, but not from its isogenic mutant in *cprA*, suggesting that lipids extracted from OMVs are as toxic to eukaryotic cells as whole OMVs. Although the lipids responsible for the anti‐autophagic activity of OMVs have not yet been identified, we confirmed here that the component responsible for this activity is present in the lipidic fraction. This result, together with the in silico analysis of the conserved enzymatic domain of HlyF and CprA, strongly supports the hypothesis that this new family of SDRs modifies lipid(s) of the bacterial membrane leading to the production of OMVs with anti‐autophagic activities.

## 4 DISCUSSION

### 4.1 Role of CprA as a novel virulence factor of *P. aeruginosa*

We have shown that CprA can induce the formation of OMVs with specific properties, such as blocking autophagic flux and exacerbating inflammasome activation. Notably, the *P. aeruginosa* reference strain PAO1, which is commonly used in research laboratories, produces a truncated form of CprA and is unable to produce OMVs with the same properties. Clearly, all the studies that have used PAO1 alone to study the role of OMVs, inflammation or autophagy must be reinterpreted in light of the fact that the strain is not truly representative of the *P. aeruginosa* species.

OMVs produced by functional CprA‐producing *P. aeruginosa* have an effect similar to that of the autophagic inhibitor chloroquine by inhibiting autophagic flux at the lysosomal fusion stage. Previous studies have shown that treatment with chloroquine can lead to an increase in the number of bacteria within bronchial epithelial cells, a decrease in bacterial clearance from the lungs and a decrease in survival rates in mice infected with *P. aeruginosa* (Junkins et al., 2013). In addition, Mohankumar et al. (2018) showed that the inhibition of autophagy with chloroquine or 3‐methyladenine resulted in an increase in the intracellular bacterial load in human corneal epithelial cells. It is possible that *P. aeruginosa*, which produces an active form of CprA exploits the unique properties of OMVs to avoid host clearance through autophagosome degradation.

OMVs also play an important role in mediating the cytosolic localization of lipopolysaccharide (LPS) and activation of the NLRP3 inflammasome machinery through the non‐canonical pathway and thereby activating an inflammatory immune response (Finethy et al., 2017; Santos et al., 2018). Although the inflammatory response remains the primary weapon against *P. aeruginosa* in acute infections, inflammasome activation by *P. aeruginosa* can be detrimental to the host. OMVs produced by bacteria expressing *hlyF* or *cprA* do not only promote intracellular delivery of LPS and PAMPs into cells but also inhibit autophagosomal clearance of these inflammasome activators and impair negative feedback control of the inflammasome, leading to an excessive and uncontrolled inflammatory state that is detrimental to the host (David et al., 2022). Excessive NLRP3 inflammasome activation can induce dysregulated inflammation leading to multiple organ failure and death. In addition, this activation can be exhausted and contribute to post‐septic immunosuppression, leading to impaired functions of innate and adaptive immune cells (Deng et al., 2018; Hotchkiss et al., 2016; Park et al., 2010; Peng et al., 2020; Shah et al., 2012). The increased inflammatory response is most likely responsible for the survival advantage of mice infected with the *cprA* mutant strain compared to the PA14 wild strain of *P. aeruginosa* (Figure 5b). The fact that CprA also played a role in antimicrobial resistance (Gutu et al., 2015) is in line with recent studies showing that certain resistance mechanisms may contribute to increased pathogenic capacity, contrary to what was previously accepted (Jordana‐Lluch et al., 2023).

### 4.2 Pathoadaptation and expression of *cprA*

The activation of PmrAB after exposure to polymyxin B and antimicrobial peptides is followed up by the activation of expression of the PmrAB regulon, including *cprA* (Mcphee et al., 2006; McPhee et al., 2003; Moskowitz et al., 2004). We have demonstrated that the pro‐inflammatory activity of OMVs from *P. aeruginosa* producing an active form of CprA is dependent on the activation of the two‐component system PmrAB (Figure 4), consistently with the fact that PmrB is the main regulator of *cprA* (Mcphee et al., 2006). Independently of the two‐component system PmrAB regulation, *cprA* has been identified to be upregulated in the presence of antibiotics such as polymyxin B, colistin or tobramycin (Cianciulli Sesso et al., 2021; Murray et al., 2015). Altogether, this suggests that antibiotic treatments during *P. aeruginosa* infection may have a significant impact on the exacerbation of *P. aeruginosa* infection, via upregulation of *cprA*, notably via the activation of the PmrAB two‐component system.

In addition to the activation of PmrAB, exposure to antibiotics such as polymyxin B leads to the emergence of different missense mutations in *pmrB*, called ‘gain‐of‐function’ mutations, which cause the activation of the PmrAB regulon regardless of the presence of activating signals in the environment (Han et al., 2019; Moskowitz et al., 2004). As the two‐component system, PmrAB activates the expression of genes involved in antibiotic resistance, these mutations have been associated with increased resistance to antimicrobial peptides and antimicrobial cationic peptides in *P. aeruginosa* (Bolard et al., 2019; Moskowitz et al., 2012). Here we have shown that a gain‐of‐function allele of *pmrB* increased the production of toxic OMVs blocking the autophagic flux and may consequently exacerbate the inflammasome activation and increase virulence.

In contrast to ‘gain‐of‐function’ mutations, some mutations in PmrB, known as ’loss‐of‐function’ mutations, may contribute to chronic infections by promoting biofilm formation (Hasan et al., 2022) or facilitating colonization and adhesion to the airway epithelium (Bricio‐Moreno et al., 2018). Given that we have shown that strains with deleted *pmrAB* no longer produce harmful OMVs, it is likely that loss‐of‐function mutations in PmrB result in the drastic reduction of CprA production. It is worth considering that this may be a strategy for *P. aeruginosa* to reduce the production of pro‐inflammatory OMVs during chronic infection, which could lead to a decrease in virulence and allow the bacteria to avoid host cell destruction. This hypothesis is supported by the work of Phuong et al. (2021).

### 4.3 Unveiling of a novel family of virulence factors

There is a large number of highly diverse species of Gram‐negative bacteria with a gene in their genome encoding an ortholog of CprA and HlyF. These orthologs are distributed in at least two classes of bacteria, the *Gammaproteobacteria* and the *Betaproteobacteria*. In these two classes of bacteria, we have shown that the gene for these orthologs enables the production of a protein in *E. coli* that is able to induce the production of OMVs blocking the autophagic flux. The majority of these orthologs are located on the bacterial chromosome and are probably part of the ‘core genome’. It is likely that the primary function of these orthologs is not virulence, but to be involved in other functions associated with the production of OMVs such as the acquisition of nutrients, killing of other bacteria in competition for the same ecological niche, stress responses or biofilm formation (Kulp & Kuehn, 2010). However, some of these bacteria are also strict or opportunistic pathogens for humans, animals or plants such as *Y. pestis*, *Y. pseudotuberculosis*, *K. pneumoniae*, *P. aeruginosa, R. solaneacearum* and *D. dadantii* amongst others (Figure 6). The hyperinflammatory nature of the OMVs produced by these bacteria could clearly be a challenge to the host during acute systemic infections. It is reasonable to assume that the prognosis of a systemic infection would be worse if caused by a Gram‐negative bacterium that produces CprA, HlyF or one of their orthologs.

The toxicity observed in OMVs from non‐pathogenic bacteria, such as laboratory strains of *E. coli*, that produce HlyF or CprA, cannot be attributed to the inherent toxicity of these cytoplasmic SDRs. Rather, it is caused by their enzymatic activity on substrates that are conserved amongst bacteria belonging to the classes of *Gammaproteobacteria* and *Betaproteobacteria*. Lipids are potential candidates, but this hypothesis is currently based on an in silico analysis of the structure and domains of these SDRs. This study initiates an exploration into a new family of virulence determinants within Gram‐negative bacteria, which opens up new avenues for research and therapeutic interventions.

The expression of *hlyF* and *cprA* is controlled by PhoPQ and PmrAB respectively, two conserved TCS responding to a low magnesium environment and antibiotic treatment to control virulence expression in Gram‐negative pathogens (Groisman et al., 2021). The fact that the expression of these SDRs can be induced by antibiotic treatments reinforces the need to find ways to limit the expression of this new class of virulence factors.

## AUTHOR CONTRIBUTIONS

**Audrey Goman**: Conceptualization (lead); formal analysis (equal); investigation (lead); validation (lead); visualization (lead); writing ‐ original draft (lead); writing—review and editing (lead). **Bérengère Ize**: Conceptualization (equal); investigation (equal); project administration (equal); resources (equal); supervision (equal); validation (equal); writing—original draft (equal); writing—review and editing (equal). **Katy Jeannot**: Conceptualization (equal); formal analysis (equal); investigation (equal); project administration (equal); resources (equal); supervision (equal); validation (equal); writing—original draft (equal); writing—review and editing (equal). **Camille Pin**: Investigation (Equal); writing‐review and editing(Equal). **Delphine Payros**: Conceptualization (equal); formal analysis (equal); investigation (equal); project administration (equal); validation (equal); visualization (lead); writing—original draft (equal); writing—review and editing (equal). **Cécile Goursat**: Formal analysis (equal); investigation (equal); visualization (lead). **Léa Ravon‐Katossky**: Investigation (equal). **Kazunori Murase**: Formal analysis (equal); investigation (equal); project administration (equal); resources (equal); validation (equal); visualization (lead); writing—original draft (equal); writing—review and editing (equal). **Camille V. Chagneau**: Investigation (equal); resources (equal); writing—review and editing (equal). **Hélène Revillet**: Resources (equal); writing—review and editing (equal). **Frédéric Taieb**: Formal analysis (equal); funding acquisition (equal); writing—review and editing (equal). **Sophie Bleves**: Conceptualization (equal); project administration (equal); resources (equal); validation (equal); writing—review and editing (equal). **Laure David**: Formal analysis (equal); investigation (equal); validation (equal); visualization (equal); writing—review and editing (equal). **Etienne Meunier**: Conceptualization (equal); formal analysis (equal); project administration (equal); resources (equal); supervision (equal); validation (equal); visualization (equal); writing—original draft (equal). **Priscilla Branchu**: Conceptualization (lead); formal analysis (equal); funding acquisition (equal); investigation (equal); project administration (equal); supervision (lead); validation (lead); visualization (equal); writing—original draft (lead); writing—review and editing (lead). **Eric Oswald**: Conceptualization (lead); formal analysis (equal); funding acquisition (lead); project administration (equal); resources (equal); supervision (lead); validation (lead); visualization (equal); writing—original draft (lead); writing—review and editing (lead).

## CONFLICT OF INTEREST STATEMENT

The authors have declared that no competing interests exist.

## Supporting information



Supporting Information

Supporting Information

Supporting Information

Supporting Information

Supporting Information
