# Supplementary figures and images for: Uncovering a new family of conserved virulence factors that promote the production of host‐damaging outer membrane vesicles in gram‐negative bacteria
Source: J Extracell Vesicles. 2025 Jan 22;14(1):e270032. doi: 10.1002/jev2.70032 (PMC11752146; doi:10.1002/jev2.70032)

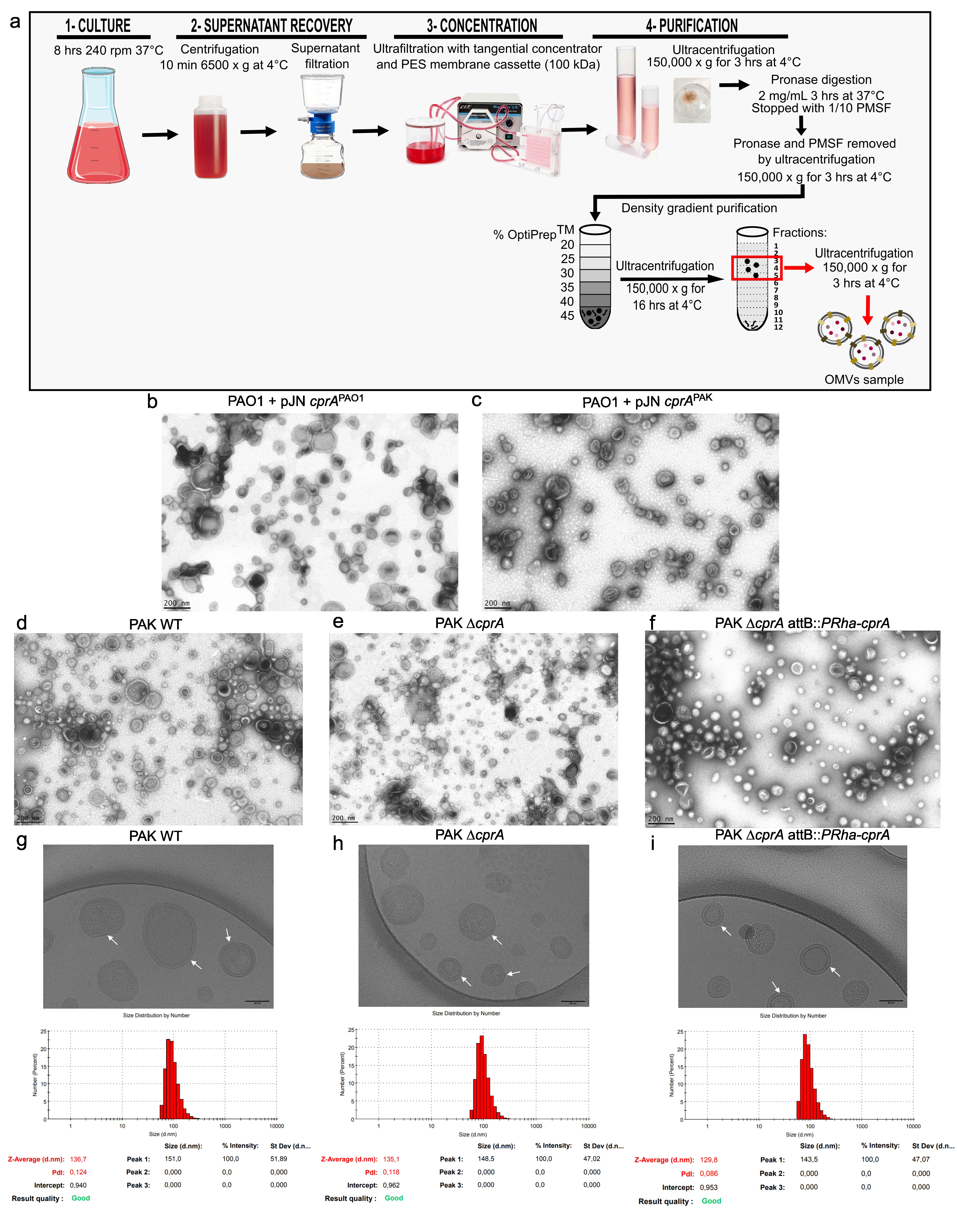

Supplement: Supplementary file 1 — Supporting Information [file JEV2-14-e270032-s004.jpg]

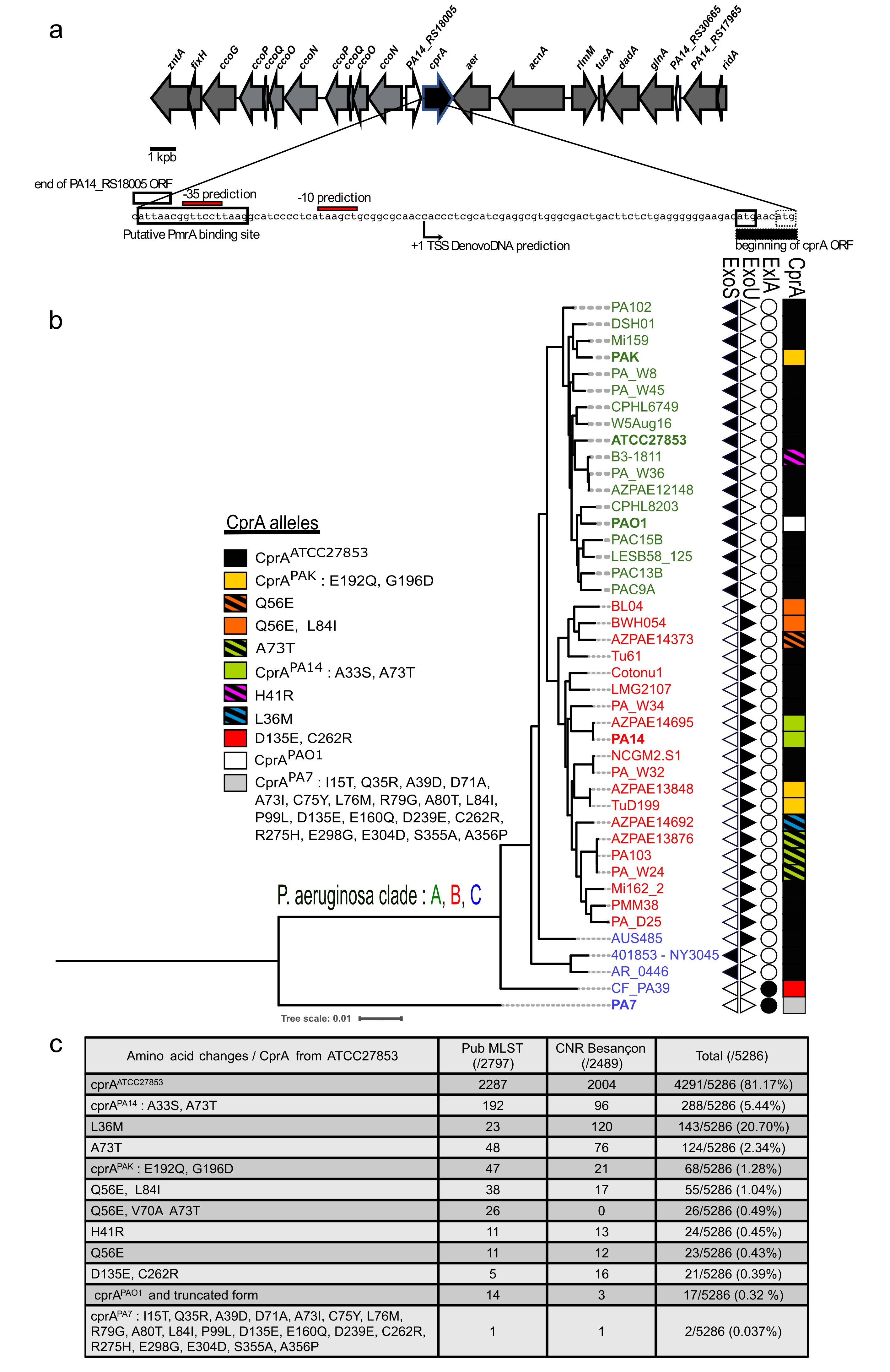

Supplement: Supplementary file 2 — Supporting Information [file JEV2-14-e270032-s001.jpg]

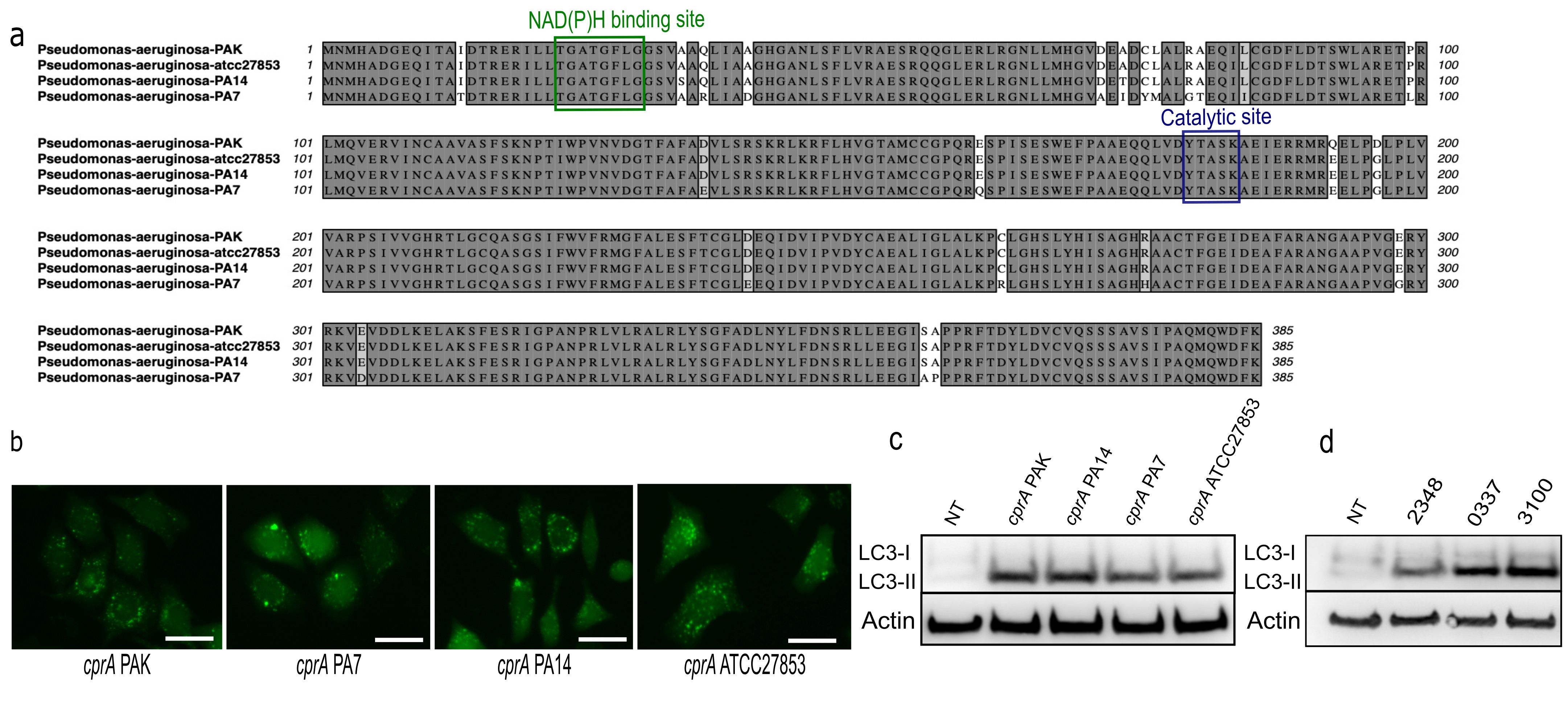

Supplement: Supplementary file 3 — Supporting Information [file JEV2-14-e270032-s002.jpg]

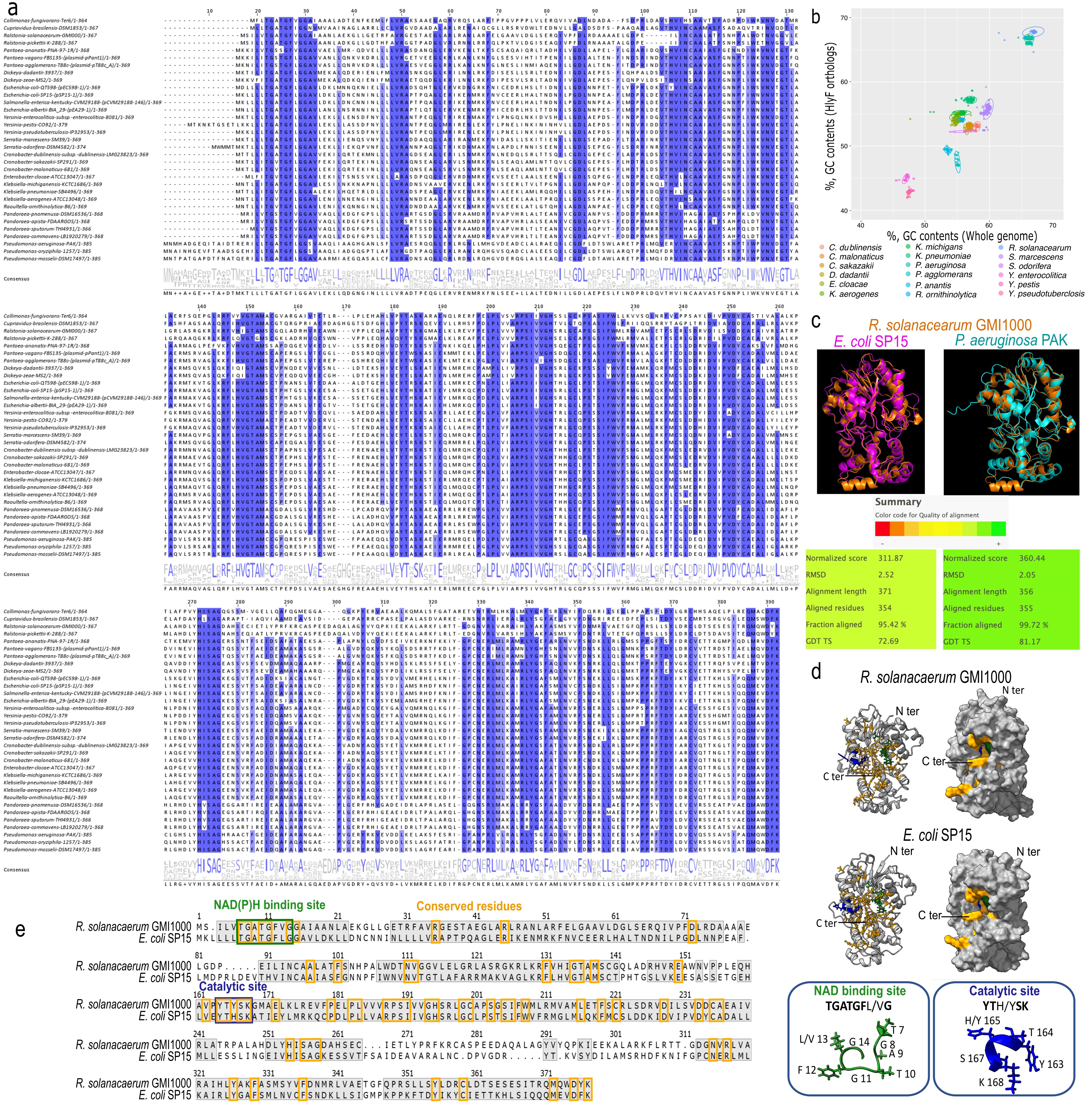

Supplement: Supplementary file 4 — Supporting Information [file JEV2-14-e270032-s005.jpg]
