# Supplementary material for: Uncovering a new family of conserved virulence factors that promote the production of host‐damaging outer membrane vesicles in gram‐negative bacteria
Source: J Extracell Vesicles. 2025 Jan 22;14(1):e270032. doi: 10.1002/jev2.70032 (PMC11752146; doi:10.1002/jev2.70032)
Supplement: Supplementary file 5 — Supporting Information [file JEV2-14-e270032-s003.docx]

**Supplementary Table 1. Strains and plasmids used in this study.**

| **Strains** | | **Relevant characteristics** | **Reference** | | |
| --- | --- | --- | --- | --- | --- |
| *E. coli* BL21 (DE3) | |  | Sigma, CMC0014 | | |
| *E. coli* SP15 | |  | Johnson et al., 2002 | | |
| *E. coli* CC118λpir | | Use to propagate pKNG101 derivatives | Sophie Bleves’ laboratory collection | | |
| *E. coli* SM10 | | Use to propagate SM10 to propagate Mini-CTX1 derivatives | Sophie Bleves’ laboratory collection | | |
| *E. coli* DH5α | |  | Common laboratory strain | | |
| *P. aeruginosa* PAO1 | |  | Sophie Bleves’ laboratory collection | | |
| *P. aeruginosa* PAK | |  | Sophie Bleves’ laboratory collection | | |
| *P. aeruginosa* PA7 | |  | Imbert et al., 2017 | | |
| *P. aeruginosa* ATCC 27853 | |  | ATCC | | |
| *P. aeruginosa* PAK ∆*cprA* | | Δ*cprA* | This study | | |
| *P. aeruginosa* PAK ∆*cprA attB::PRha-cprA* | | PAK with a chromosomal miniCTX1-rhaSR-PrhaBAD::cprA, Tc^R^ | This study | | |
| *P. aeruginosa* PA14 | |  | Sophie Bleves’ laboratory collection | | |
| *P. aeruginosa* PA14 ∆*cprA* | | Δ*cprA* | This study | | |
| *P. aeruginosa* PA14 ∆*cprA attB::PRha-cprA* | | PA14 with a chromosomal miniCTX1-rhaSR-PrhaBAD::cprA, Tc^R^ | This study | | |
| *P. aeruginosa* PA14 ∆pmrAB | | ∆pmrAB | This study | | |
| *P. aeruginosa* 2348 | | Isolated from cystic fibrosis patients treated with colistimethate sodium aerosol | University hospitals of Toulouse | | |
| *P. aeruginosa* 0337 | | Isolated from infection caused by medical device | University hospitals of Toulouse | | |
| *P. aeruginosa* 3100 | | Isolated from ear nose throat infection | University hospitals of Toulouse | | |
|  | |  |  | | |
|  | |  |  | | |
| **Plasmids** | **Relevant characteristics** | | | **Reference** |  |
| pK184 |  | | | Jobling & Holmes, 1990 |  |
| pAGO-15 | pK184 vector encoding the 6His-*hlyF* fusion protein under *hlyF* promoter | | | This study |  |
| pAGO-16 | pK184 vector encoding the 6His-*hlyF*SDM fusion protein under *hlyF* promoter | | | This study |  |
| pAGO-25 | pK184 vector encoding the 6His-*cprA^PA7^* fusion protein under *hlyF* promoter | | | This study |  |
| pAGO-30 | pK184 vector encoding the 6His-*cprA^PAO1^* fusion protein under *hlyF* promoter | | | This study |  |
| pAGO-31 | pK184 vector encoding the 6His-*cprA^PA14^* fusion protein under *hlyF* promoter | | | This study |  |
| pAGO-32 | pK184 vector encoding the 6His-*cprA^PAK^* fusion protein under *hlyF* promoter | | | This study |  |
| pAGO-41 | pK184 vector encoding the 6His-*cprA^ATCC27853^* fusion protein under *hlyF* promoter | | | This study |  |
| pAGO-29 | pK184 vector encoding the 6His-*HlyF-like from* *R. solanacearum* GMI1000 fusion protein under *hlyF* promoter | | | This study |  |
| pAGO-19 | pK184 vector encoding the 6His-*HlyF-like from* *K. pneumoniae* SB4496 fusion protein under *hlyF* promoter | | | This study |  |
| pAGO-21 | pK184 vector encoding the 6His-*HlyF-like from* *K. aerogenes* ATCC13048 fusion protein under *hlyF* promoter | | | This study |  |
| pAGO-17 | pK184 vector encoding the 6His-*HlyF-like from* *E. cloacae* ATCC13047 fusion protein under *hlyF* promoter | | | This study |  |
| pAGO-23 | pK184 vector encoding the 6His-*HlyF-like from* *S. marcescens* SM39 fusion protein under *hlyF* promoter | | | This study |  |
| pAGO-53 | pK184 vector encoding the 6His-*HlyF-like from* *Y. pestis* KIM6+ *pla-* fusion protein under *hlyF* promoter | | | This study |  |
| pAGO-55 | pK184 vector encoding the 6His-*HlyF-like from* *Y. pseudotuberculosis* IP32953 fusion protein under *hlyF* promoter | | | This study |  |
| pAGO-42 | pK184 vector encoding the 6His-*HlyF-like from* *P. gingivalis* ATCC33277 fusion protein under *hlyF* promoter | | | This study |  |
| pJN105 | Cloning vector with P_BAD_ for 6His-*cprA* expression | | | Newman et al., 1999 |  |
| pJN *cprA*^PAO1^ (pAGO-37) | pJN105 vector encoding the 6His-*cprA^PAO1^* fusion protein | | | This study |  |
| pJN *cprA*^PAK^ (pAGO-38) | pJN105 vector encoding the 6His-*cprA^PAK^* fusion protein | | | This study |  |
| pKNG101 | Suicide vector Sm^R^; *sacB+* | | | Kaniga et al., 1991 |  |
| pRK2013 | Helper vector, Tra+, Mob+, ColE1, Km^R^ | | | Sophie Bleves’ laboratory collection |  |
| pKNG*cprA* | Suicide vector for *cprA* deletion in PAK, Sm^R^ | | | This study |  |
| miniCTX1-*rha*SR-*Prha*BAD | Integration expression vector. Contains *attP* site for integration at the *attB* site of *P. aeruginosa* chromosome; Tc^R^ | | | Meisner & Goldberg, 2016 |  |
| miniCTX-*PRha*-*cprA* | Integration expression vector for *cprA* expression, Tc^R^ | | | This study |  |
| miniCTX-1 | Self-proficient integration vector, Ω-FRT-*att*P-MCS, ori, int, oriT ; Tc^R^ | | | Hoang et al., 2000 |  |
| miniCTX-*cprA* ^PA14^ | Integration expression vector for *cprA* expression, Tc^R^ | | | This study |  |
| pME6012 | Broad-host-range expression plasmid; Tet^R^ | | | Bolard et al., 2019 |  |
| pABWT | pME6012 carrying genes *pmrAB* from strain PAO1 | | | Bolard et al., 2019 |  |
| pAB16.2 | pME6012 carrying genes *pmrAB* from PAO1 AB16.2 (with PmrB ∆172) | | | Bolard et al., 2019 |  |
| pKNGΔpmrAB | Suicide vector for *pmrAB* deletion in PA14, Sm^R^ | | | This study |  |
|  | |  |  | | |

**Supplementary Table 2. List of primers used in the study**

| **Primer** | **Sequence** | **Use** | **Reference** |
| --- | --- | --- | --- |
| PB13 | CGGAATTCCCTGAAGCATGCCTGTTTTTAAC | Amplification of *hlyF* promoter | This study |
| AGO63 | ATCTAACCTCTTTTGTTTTCATGTGATC | Amplification of *hlyF* promoter | This study |
| AGO51 | GGGTACCGATGACGACGACAAGATGAAATTATTATTACTTACAGGTGC | Amplification of *hlyF* ORF | This study |
| HLYF EC DOWN SALI | GTCGACTTATTTAAAATCAACTTCCA | Amplification of *hlyF* ORF | This study |
| AGO64 P2 BIS | CATGAAAACAAAAGAGGTTAGATATGCACCATCATCATCATCA | Amplification of 6-His and S-tag | This study |
| AGO50 | CTTGTCGTCGTCATCGGTAC | Amplification of 6-His and S-tag reverse // pAGO-15 linearization | This study |
| AGO83 | GTCGACCTGCAGGCATGC | pAGO-15 linearization | This study |
| PB263 | CAGATATTCTATTGTTGCTGCTGAATGCGTAAACTCCACTAAATGTTCACCTGTTTCTGA | Antisense primer for site-directed mutagenesis of HlyF catalytic site | This study |
| PB264 | TCAGAAACAGGTGAACATTTAGTGGAGTTTACGCATTCAGCAGCAACAATAGAATATCTG | Sense primer for site-directed mutagenesis of HlyF catalytic site | This study |
| AGO76 | TGGGTACCGATGACGACGACAAGATGAACATGCATGCCG | Amplification of *cprA* allele of PAK, PAO1, PA14, PA7 and ATCC27853 strains | This study |
| AGO84 | AGCTTGCATGCCTGCAGGTCGACTCACTTGAAGTCCCATTGC | Amplification of *cprA* allele of PAK, PAO1, PA14 and ATCC27853 strains | This study |
| AGO77 | AGCTTGCATGCCTGCAGGTCGACTCACTTGAAATCCCACTGC | Amplification of *cprA* allele of PA7 strain | This study |
| AGO87 | TGGGTACCGATGACGACGACAAGATGAGTATTCTGGTCACCGGCG | Amplification of HlyF-like orthologous from *R. solanacearum* GMI1000 | This study |
| AGO88 | AGCTTGCATGCCTGCAGGTCGACCTATTTGTAGTCCCACTGCATCTGC | Amplification of HlyF-like orthologous from *R. solanacearum* GMI1000 | This study |
| AGO52 | TGGGTACCGATGACGACGACAAGATGTTATTTATTACAGGCGTGAC | Amplification of HlyF-like orthologous from *E. cloacae* ATCC13047 | This study |
| AGO82 | AGCTTGCATGCCTGCAGGTCGACTTATTTGAAATCAACCGCCATTTG | Amplification of HlyF-like orthologous from *E. cloacae* ATCC13047 | This study |
| AGO75 | TGGGTACCGATGACGACGACAAGATGAAAACGTTACTGTTGACCGG | Amplification of HlyF-like orthologous from *S. marcescens* SM39 | This study |
| AGO74 | AGCTTGCATGCCTGCAGGTCGACTTATTTGAAGTCCACCACCATC | Amplification of HlyF-like orthologous from *S. marcescens* SM39 | This study |
| AGO78 | TGGGTACCGATGACGACGACAAGATGAATACATTATTAATTACAGGCGTTACC | Amplification of HlyF-like orthologous from *K. aerogenes* ATCC13048 | This study |
| AGO79 | AGCTTGCATGCCTGCAGGTCGACTTACTTAAAATCGACGGCCATCTG | Amplification of HlyF-like orthologous from *K. aerogenes* ATCC13048 | This study |
| AGO80 | TGGGTACCGATGACGACGACAAGATGACCACATTATTAATTACCGGCGTTACG | Amplification of HlyF-like orthologous from *K. pneumoniae* SB4496 | This study |
| AGO81 | AGCTTGCATGCCTGCAGGTCGACTTATTTAAAGTCGACGGCCATTTG | Amplification of HlyF-like orthologous from *K. pneumoniae* SB4496 | This study |
| AGO136 | TGGGTACCGATGACGACGACAAGATGAAAAAGCTTTTATTAACCG | Amplification of HlyF-like orthologous from *Y. pseudotuberculosis* IP32953 & *Y. pestis* KIM6+ *pla-* | This study |
| AGO137 | AGCTTGCATGCCTGCAGGTCGACTTATTTAAAATCGACAGCCATTTG | Amplification of HlyF-like orthologous from *Y. pseudotuberculosis* IP32953 & *Y. pestis* KIM6+ *pla-* | This study |
| AGO106 | TGGGTACCGATGACGACGACAAGATGGGGAACAAGCGAGTTCTCAT | Amplification of HlyF-like orthologous from  *P. gingivalis* ATCC33277 | This study |
| AGO107 | AGCTTGCATGCCTGCAGGTCGACCTAACGACGGATTTGTCCGGTG | Amplification of HlyF-like orthologous from  *P. gingivalis* ATCC33277 | This study |
| AGO96 | GCACTGCAGATGCACCATCATCATCATCATTC | Amplification of the fusion protein 6-His S-tag *cprA* allele from PAK, PAO1, PA14 and PA7 strains for cloning within PstI restriction site | This study |
| AGO90 | GCAGAGCTCTCACTTGAAGTCCCATTGC | Amplification of the fusion protein 6-His S-tag *cprA* allele from PAK and PAO1 strains for cloning within SacI restriction site | This study |
| AGO92 | GCAGAGCTCTCACTTGAAGTCCCATTGCATC | Amplification of the fusion protein 6-His S-tag *cprA* allele from PA14 for cloning within SacI restriction site | This study |
| AGO91 | GCAGAGCTCTCACTTGAAATCCCACTGC | Amplification of the fusion protein 6-His S-tag *cprA* allele from PA7 for cloning within SacI restriction site | This study |
| cprAupFor | ggtcgacggatccccgggGCGCTTCGAGTTTCCCTA | Amplification of 500 bp upstream cprA from PAK | This study |
| cprAupRev | TGCCGTCACATGTCTTCCCCCCTCAGAGA | Amplification of 500 bp upstream cprA from PAK | This study |
| cprAdownFor | AAGACATGTGACGGCAGGCGCGGGGAGCT | Amplification of 500 bp downstream cprA from PAK | This study |
| cprAdownRev | tgcatccgcgggcccgggCGACCAGACCAACCTGCT | Amplification of 500 bp downstream cprA from PAK | This study |
| cprArhaup | CAGGAATTCCTCGAGAAGCTTAAGAGGTTAGATATGCAC | Amplification *cprA* gene along with Nterminal His- and S-tags from pAGO-32 | This study |
| cprArhadown | GTCGACGGTATCGATAAGCTTTCACTTGAAGTCCCATTG | Amplification *cprA* gene along with Nterminal His- and S-tags from pAGO-32 | This study |
| PCR-ipmrABC1 | TGTCGAACTGACCCAGCTAC | Amplification of upstream pmrA and pmrB from PA14 | This study |
| PCR-ipmrABC2 | CTTCCAGGTCACCCATTCCACGGTATC | Amplification of upstream pmrA and pmrB from PA14 | This study |
| PCR-ipmrABC3 | GGGTGACCTGGAAGTGCAGGTGTTCCT | Amplification of downstream pmrA and pmrB from PA14 | This study |
| PCR-ipmrABC4 | GTGCTGAGCTCCTCGATCTT | Amplification of downstream pmrA and pmrB from PA14 | This study |

**Supplementary Table 3. Clinical scoring**

**Supplementary Table 4.**  **Summary of Pangenome profile of 18 bacterial species with HlyF orthologs**

| **Species** | **No. of strain** | **Pan-genome profile** | | | **Distribution of  HlyF ortholog** |
| --- | --- | --- | --- | --- | --- |
|  |  | **core*** | **dispensable** | **unique** |  |
| *Cronobacter dublinensis* | 38 | 2157 | 5857 | 5624 | 100 (38/38) |
| *Cronobacter malonaticus* | 45 | 2313 | 5247 | 4634 | 93.3 (42/45) |
| *Cronobacter sakazakii* | 368 | 1224 | 15003 | 10562 | 100 (368/368) |
| *Dickeya dadantii* | 13 | 1957 | 3586 | 1909 | 84.6 (11/13) |
| *Enterobacter cloacae* | 81 | 2486 | 10418 | 8180 | 98.8 (80/81) |
| *Klebsiella aerogenes* | 258 | 1837 | 15026 | 14631 | 99.6 (257/258) |
| *Klebsiella michiganensis* | 163 | 2701 | 20857 | 14504 | 100 (163/163) |
| *Klebsiella pneumoniae* | 184 | 2159 | 13222 | 7719 | 100 (184/184) |
| *Pantoea agglomerans* | 78 | 2650 | 8053 | 7435 | 37.2 (29/78)** |
| *Pantoea ananatis* | 56 | 2911 | 6920 | 5809 | 100 (56/56) |
| *Pseudomonas aeruginosa* | 820 | 2780 | 23978 | 11760 | 100 (820/820) |
| *Ralstonia solanacearum* | 117 | 1389 | 11479 | 8230 | 97.4 (114/117) |
| *Raoultella ornithinolytica* | 69 | 3204 | 9440 | 9152 | 100 (69/69) |
| *Serratia marcescens* | 425 | 1566 | 17079 | 11365 | 100 (425/425) |
| *Serratia odorifera* | 6 | 3987 | 1092 | 1575 | 100 (6/6) |
| *Yersinia enterocolitica* | 176 | 1870 | 11443 | 6510 | 100 (176/176) |
| *Yersinia pestis* | 400 | 604 | 7089 | 5779 | 100 (400/400) |
| *Yersinia pseudotuberculosis* | 74 | 2168 | 6228 | 3501 | 100 (74/74) |

***, present in 100% of the isolates; **, include Plasmid-encoded**
